# Side‐on Coordination in Isostructural Nitrous Oxide and Carbon Dioxide Complexes of Nickel

**DOI:** 10.1002/anie.202011301

**Published:** 2021-02-17

**Authors:** Braulio M. Puerta Lombardi, Chris Gendy, Benjamin S. Gelfand, Guy M. Bernard, Roderick E. Wasylishen, Heikki M. Tuononen, Roland Roesler

**Affiliations:** ^1^ Department of Chemistry University of Calgary 2500 University Drive NW Calgary AB T2N 1N4 Canada; ^2^ Gunning-Lemieux Chemistry Centre University of Alberta 11227 Saskatchewan Drive NW Edmonton AB T6G 2G2 Canada; ^3^ Department of Chemistry, Nanoscience Centre University of Jyväskylä, P.O. Box 35 FI-40014 Jyväskylä Finland

**Keywords:** back bonding, carbon dioxide, N-heterocyclic carbenes, nickel, nitrous oxide

## Abstract

A nickel complex incorporating an N_2_O ligand with a rare η^2^‐*N*,*N*′‐coordination mode was isolated and characterized by X‐ray crystallography, as well as by IR and solid‐state NMR spectroscopy augmented by ^15^N‐labeling experiments. The isoelectronic nickel CO_2_ complex reported for comparison features a very similar solid‐state structure. Computational studies revealed that η^2^‐N_2_O binds to nickel slightly stronger than η^2^‐CO_2_ in this case, and comparably to or slightly stronger than η^2^‐CO_2_ to transition metals in general. Comparable transition‐state energies for the formation of isomeric η^2^‐*N*,*N*′‐ and η^2^‐*N*,*O*‐complexes, and a negligible activation barrier for the decomposition of the latter likely account for the limited stability of the N_2_O complex.

## Graphical abstract

The characterization of η^2^‐*N*,*N*′‐N_2_O and CO_2_ complexes of nickel and the associated computational study reveal that the bonding ability of N_2_O to nickel is intermediate between that of CO_2_ and that of H_2_C=CH_2_. It is shown that in general, N_2_O η^2^‐binds to metals comparably to or stronger than CO_2_, indicating that the rarity of η^2^‐N_2_O metal complexes is due mostly to its oxidizing character and not to its weak σ‐donating and π‐accepting properties.

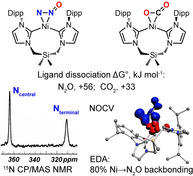

Among the numerous oxides of nitrogen, nitrous oxide (N_2_O) is most intimately intertwined with modern human activities. It figures on the WHO's List of Essential Medicines for use in pain management,^[1]^ and it also has a long history as a recreational drug dubbed “laughing gas”.^[2]^ It is used as an oxidant (“nitrous”) in racing engines and is a suitable propellant in rockets,^[3]^ as well as in whipped cream and cooking oil canisters. Industrially, N_2_O is an important by‐product in nitric acid and adipic acid manufacturing.^[4]^ Although industrial pollutants are not to be neglected, natural, enzymatic denitrification processes^[5]^ are the main source of N_2_O in the environment and for this reason the gas was proposed to be part of the biosignature of life on exoplanets.^[6]^ The widespread use of nitrogen fertilizers led to an enhancement of denitrification processes and N_2_O rose to prominence as a greenhouse gas 300 times more potent than CO_2_, and “the dominant ozone‐depleting substance emitted in the 21^st^ Century”.^[7]^ Although its decomposition into elements is thermodynamically favorable (Δ_f_ *H*°_gas_ 82.1 kJ mol^−1^), the high activation barrier (250 kJ mol^−1^)^[8]^ associated with this process means that N_2_O persists in the atmosphere for an average of 117(8) years.^[9]^ Consequently, interest towards using N_2_O as a synthon,^[10]^ as well as towards catalyzing its decomposition into elements has increased in recent years,^[11]^ in turn prompting investigations meant to elucidate the interaction of this prominent small molecule with metals.

N_2_O reacts readily with numerous metal complexes and organic substrates, mostly as an oxidant but also as a nitrogen atom donor,[^4^, ^10^] and can be trapped by frustrated Lewis pairs^[12]^ and N‐heterocyclic carbenes (NHCs).^[13]^ Its reactivity involving insertion into M−C and M−H bonds is well documented.[^10^, ^14^] In contrast to its isoelectronic counterpart CO_2_, which has a rich coordination chemistry,^[15]^ N_2_O has been generally described as a poor, or exceedingly poor ligand due to its weak σ‐donating and π‐accepting properties, low polarity, and oxidizing character.[^8b^, ^16^]

Extensive investigations of ruthenium derivative **A** (Figure 1), which remained for more than three decades the only known metal complex of nitrous oxide, revealed that N_2_O coordinated in a linear fashion via the terminal nitrogen and was a poor ligand susceptible to reduction and displacement.^[17]^ These conclusions were supported by computational studies,[^17c^, ^18^] the spectroscopic characterization of complex **B**,^[19]^ as well as the NMR characterization of surface‐coordinated N_2_O.^[20]^ Confirmation of these findings was provided over the last decade by the comprehensive characterization of discrete, end‐on bonded complexes **C**, **D** and **E** (Figure 1).[^21^, ^22^, ^23^] The rich coordination chemistry of CO_2_ suggests that the isoelectronic N_2_O molecule should also be able to adopt a bent, *N*,*N*′‐side‐on coordination mode, which had been probed computationally for surface binding.^[24]^ Linear N_2_O bound at the [4Cu:2S] active site of nitrous oxide reductase has been shown to display long, side‐on Cu⋅⋅⋅N contacts.^[25]^ Ultimately, the first η^2^‐*N*,*N*′‐N_2_O complex **F**, which was persistent below −25 °C, was recently characterized and the π‐basicity of the metal was shown to be key to its isolation.^[26]^

**Figure 1:**
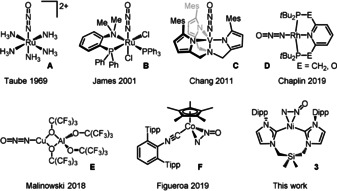
Transition metal complexes of N_2_O.

We reported on a bis(NHC)_2_Ni^0^‐GeCl_2_ complex incorporating a siloxane‐linked (NHC)_2_Ni^0^ fragment with a bent L_2_M geometry.^[27]^ Computational studies indicated that this fragment featured the frontier orbitals necessary for efficient η^2^‐interactions with π‐acidic ligands.[^18c^, ^28^] Thus, we hypothesized that a bis(NHC)_2_ supported Ni^0^ would be an excellent candidate for stabilizing side‐on, η^2^‐N_2_O complexes, especially taking into account the resilience of NHC ligands to oxidation. Design of ligand **1**, incorporating a shorter silane linker, aimed to impose a narrow C_NHC_‐Ni‐C_NHC_ angle and increase the π‐basicity of the metal.^[29]^ This allowed us to characterize analogous η^2^‐bound Ni^0^ complexes of N_2_O and CO_2_ and to assess the relative binding ability of the two ligands for the first time.

Prepared by deprotonation of its bis(imidazolium) precursor, ligand **1** reacted with Ni(cod)_2_ to yield (**1**)Ni(η^2^‐cod), **2** (Scheme 1). Solution ^1^H NMR analysis of **2** revealed a broad, complex spectrum denoting *C*_1_ symmetry, reflected in the ^13^C NMR spectrum by the presence of two resonances for the coordinated carbene carbons (200.4 and 208.4 ppm). Reduced conformational fluxionality in complexes containing bis(NHC)Ni fragments was shown to lead to broad, poorly resolved resonances in the solution NMR spectra, as well as lowering of the expected time‐averaged symmetry.^[27]^ An X‐ray diffraction experiment on **2** confirmed chelation of the ligand to Ni in a bent geometry (C1‐Ni1‐C8 107.9(1)°) (Figure S28) and the η^2^‐coordination of 1,5‐cyclooctadiene.

**Scheme 1:**
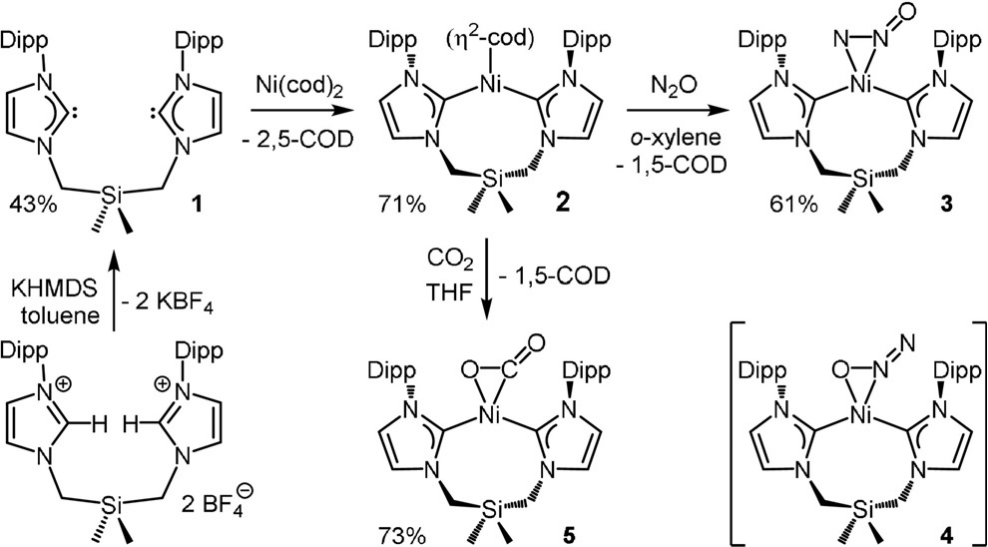
Synthesis of compounds **1**–**3** and **5**, and the postulated, fleeting η^2^‐*N*,*O*‐isomer **4**. Dipp=2,6‐diisopropylphenyl.

In solution, complex **2** reacted with 1 atm of N_2_O at room temperature to yield **3**, which was isolated as a yellow crystalline solid. The low solubility of **3** precluded its characterization in solution. As a solid, it can be stored for months at −78 °C and handled at room temperature in vacuum or under an inert atmosphere, but partial decomposition is apparent after 12 hours at room temperature (by IR). Heating to 70 °C in THF leads to dissolution upon N_2_ development (Figure S3). The ^13^C CP‐MAS NMR spectrum of **3** (Figure S19) features two resonances corresponding to the coordinated carbene carbons at 183.7 and 192.5 ppm, similar to the values measured in solution for **2**.

The ^15^N CP‐MAS NMR resonances for bound N_2_O in an isotopically enriched sample of **3** were observed at 365 and 312 ppm (Figure 2), corresponding to the central and terminal nitrogen atoms in N_2_O, respectively (vs. the gas phase computed values of 395 and 313 ppm). These values are significantly deshielded compared to those observed in the κ^1^‐*N*‐N_2_O and the η^2^‐*N*,*N*′‐N_2_O complexes, as well as those for free N_2_O (Table 1). Resonances corresponding to the naturally abundant nitrogen atoms in the imidazole rings appear between 187.5–190.4 ppm, matching literature data for NHC ligands.^[30]^ Infrared spectroscopy suggests that N_2_O is side‐on, η^2^‐*N*,*N*′‐coordinated in **3**. The observed *ν*_NN_ stretching and *ν*_NNO_ bending vibrations (Figure 3) at 1533 and 1138 cm^−1^, respectively (vs. the gas phase computed values of 1725 and 1243 cm^−1^, and the experimental values for **F** of 1624 and 1131 cm^−1^) shift to lower frequencies (1495 and 1121 cm^−1^) in ^15^N‐enriched samples of **3**. The *ν*_NN_ stretching vibration measured in **3** is the lowest value observed in N_2_O metal complexes, both κ^1^‐*N*‐N_2_O (2234–2303 cm^−1^ for *ν*_NN_ and 1150–1337 cm^−1^ for *ν*_NO_ in **A**–**E**) and η^2^‐*N*,*N*′‐N_2_O (1624 cm^−1^ for *ν*_NN_ and 1131 cm^−1^ for *ν*_NNO_ in **F**), in agreement with the high π‐basicity of the (**1**)Ni^0^ fragment and its strong interaction with the π* system of N_2_O.

**Figure 2:**
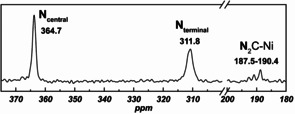
^15^N CP‐MAS NMR spectrum of **3** containing 33 % ^15^N_2_O, the latter prepared using an original method (see Supporting Information).

**Figure 3:**
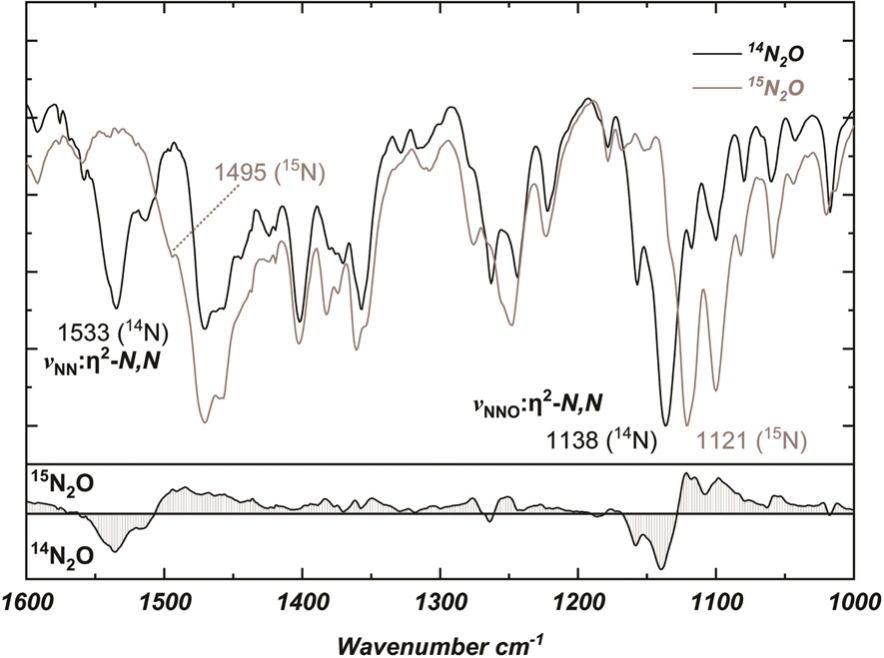
Overlaid FT‐IR spectra for **3** and **3**‐(^15^N_2_O) (99 % isotopically enriched) with spectral difference below.

**Table 1:** Selected ^15^N NMR resonances for free and bound N_2_O.

Cpd.	N_2_O^[26]^	**B** ^[19]^	**D** ^[a], [22]^	**D** ^[b], [22]^	**F** ^[26]^	**3**	**3** ^[c]^
Solv.	tol‐*d*_8_	CD_2_Cl_2_	DFB^[d]^	DFB^[d]^	tol‐*d*_8_	solid	gas
δN_term_	135	126	109	103	159	312	313
δN_cent_	218		245	246	309	365	395

[a] E=CH_2_. [b] E=O. [c] Computed. [d] DFB=1,2‐F_2_C_6_H_4_.

X‐ray crystallography revealed for **3** the expected, bent (**1**)Ni fragment (∡CNC 104.47(13)°) with the side‐on, η^2^‐*N*,*N*′‐coordinated N_2_O ligand completing the coordination sphere of nickel (Figure 4). The metric parameters characterizing the N_2_O moiety (N5−N6 1.225(4) Å and N5−O1 1.276(4) Å) are consistent with the calculated values and compare well with the N−N bond length measured in **F** (1.212(8) Å). The dihedral angle formed by the N_2_O and C_NHC_NiC_NHC_ planes measures only 8.4(3)°.

**Figure 4:**
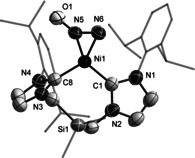
Solid‐state structure of one of the two independent molecules of **3** with 50 % thermal ellipsoids, and hydrogen atoms omitted. Selected bond lengths [Å] and angles [°] with [calculated values]: N5–N6 1.225(4) [1.210], N5–O1 1.276(4) [1.239], Ni1–N5 1.803(3) [1.821], Ni–N6 1.926(3) [1.910], Ni1–C1 1.901(3) [1.934], Ni1–C8 1.893(3) [1.919]; N5‐N6‐O1 134.7(3) [138.4], C1‐Ni1‐C8 104.47(13) [107.7].

Aiming to provide a comparison for **3**, its CO_2_ analog **5** was prepared by reaction of **2** with 1 atm of CO_2_ in THF. The product was stable under an inert atmosphere and did not dissolve in hydrocarbon or ethereal solvents. The solid‐state ^13^C NMR spectrum of **5** (Figure S21) is very similar to the spectrum of **3**, featuring two carbene resonances (188.2 and 192.2 ppm) and a resonance corresponding to the CO_2_ ligand (167.3 ppm). A characteristic *ν*_CO_ stretching vibration is observed in the IR spectrum of **5** at 1695 cm^−1^ (vs. the gas phase computed value of 1855 cm^−1^). The solid‐state structure of **5** (Figure 5) is very similar to that of **3**. The bond angles in the coordinated CO_2_ and N_2_O match closely (∡NNO 134.7(3)° in **3** vs. ∡OCO 135.0(2)° in **5**) but differences are apparent in the bond distances to their terminal atoms (N5−O1 1.275(3) Å in **3** vs. C35−O2 1.217(3) Å in **5**).

**Figure 5:**
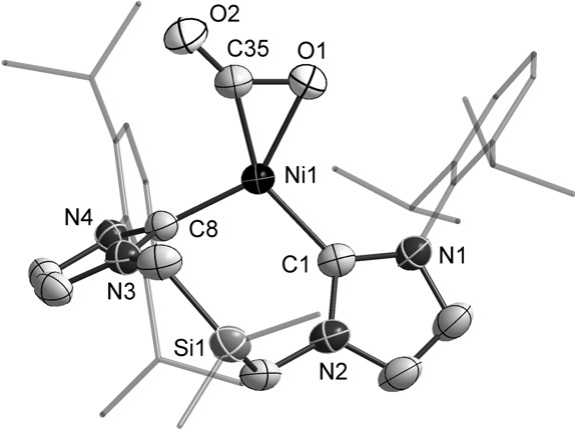
Solid‐state structure of **5** with 50 % thermal ellipsoids, and hydrogen atoms omitted. Selected bond lengths [Å] and angles [°] with [calculated values]: O1–C35 1.283(4) [1.264], C35–O2 1.218(4) [1.206], Ni1–O1 1.949(2) [1.937], Ni–C35 1.828(3) [1.839], Ni1–C1 1.973(3) [1.964], Ni1–C8 1.866(2) [1.856]; O1‐C35‐O2 134.6(3) [137.5], C1‐Ni1‐C8 106.95(10) [109.4].

A DFT comparison of the binding energies of L in (**1**)Ni(L) (with Δ*G*° in parenthesis) yielded values of 87 (33) and 110 (56) kJ mol^−1^ for L=CO_2_ and N_2_O, respectively, while for a hypothetical complex of (**1**)Ni with the classic π‐acceptor ethylene, the energies are even greater, at 129 (74) kJ mol^−1^. Furthermore, an energy decomposition analysis with ETS‐NOCV revealed that the instantaneous interaction energies of L in (**1**)Ni(L) follow a similar trend, largely owing to the significantly stronger orbital interactions (in parathesis): −401 (−643) and −415 (−709) kJ mol^−1^ for L=CO_2_ and N_2_O, respectively. The total orbital interaction term can further be decomposed using NOCV, showing a dominant contribution (83 % for **3** and 84 % for **5**) involving donation from the metal to the π* system of the ligand. Taken as a whole, the results of DFT calculations indicate that N_2_O binds to (**1**)Ni^0^ slightly stronger than CO_2_ due to stronger orbital interactions in **3**. To probe whether the observed energetic trend is more general, the equilibrium TM‐CO_2_ + N_2_O $\vec{\leftarrow }$ TM‐N_2_O + CO_2_ (TM=transition metal fragment) was analyzed computationally for 12 crystallographically characterized η^2^‐*C*,*O*‐CO_2_ complexes and their hypothetical N_2_O analogues. The data (Table S2) showed stronger binding for N_2_O in 9 systems (up to 26 kJ mol^−1^) demonstrating that when bound in η^2^‐fashion, N_2_O is a comparable or slightly better π‐acceptor than CO_2_. However, it needs to be stressed that N_2_O is oxidizing whereas CO_2_ is not, for which reason the increased binding energy in the hypothetical N_2_O systems considered above is unlikely to stabilize η^2^‐*N*,*N*‐N_2_O complexes over metal or ligand oxidation.

The energy landscape for the formation of **3** from (**1**)Ni^0^ and N_2_O was also probed with computational methods (Figure S31). The results revealed that the formation of **3** involves a modest barrier (Δ*G* ^≠^=38 kJ mol^−1^), with Δ*G*°=−56 kJ mol^−1^. The formation of the η^2^‐*O*,*N*‐N_2_O isomer, **4**, though not observed experimentally, was found to involve a greater barrier (Δ*G* ^≠^=70 kJ mol^−1^) and a minute Δ*G*° of −2 kJ mol^−1^. However, **4** appears to be a metastable species and readily converts to (**1**)NiO(N_2_) almost without a barrier. Barrierless decomposition of η^2^‐*O*,*N*‐N_2_O bound to iron has been investigated computationally and matched experimental observations.^[31]^ Thus, at a relative energy of −63 kJ mol^−1^, (**1**)NiO(N_2_) represents the lowest energy point on the potential energy surface and confirms that metal‐oxo formation is thermodynamically favored over η^2^‐*N*,*N*‐N_2_O complex formation, albeit only by 7 kJ mol^−1^. As suggested by the calculated energy landscape, **3**, unlike **5**, is a kinetic, not a thermodynamic, product, in agreement with its limited stability. Similarly, decomposition of **F** was reported to proceed via formation of a reactive metal‐oxo species and transfer of oxygen to the ancillary isocyanide ligand.^[26]^

To summarize, employing the π‐basic fragment (**1**)Ni, we isolated **3** by reaction of **2** with N_2_O. The rare η^2^‐*N*,*N*′‐coordination mode of the N_2_O ligand in **3** was proved by single‐crystal X‐ray crystallography, as well as ^15^N CP‐MAS NMR and IR spectroscopy aided by ^15^N isotopic enrichment. The isostructural, η^2^‐CO_2_ complex **5** was also synthesized, allowing a direct comparison of the metal binding properties of the two isoelectronic small molecules of environmental relevance. Computational studies indicate that π‐acceptance is the main contributor to N_2_O binding in **3**, and place the η^2^‐*N*,*N*′‐metal binding ability of this ligand to the (**1**)Ni fragment in‐between that of CO_2_ and ethylene. In general, the η^2^‐*N*,*N*′‐binding ability of N_2_O to transition metals is found to be comparable to, or slightly better than that of CO_2_. This demonstrates that the need for a strongly π‐basic metal fragment comes not so much from the frequently invoked “poor σ‐donating and π‐accepting properties” of N_2_O, but from the need to stabilize η^2^‐*N*,*N*′‐coordination over the thermodynamically more favorable metal‐oxo formation. The well‐known oxidizing character of N_2_O may be mostly, if not entirely responsible for the scarcity of η^2^‐metal complexes employing this ligand, and more of such complexes are expected to be in reach in designs featuring the right balance of π‐basicity and resilience to oxidation at the metal center and associated ligands.

## Conflict of interest

The authors declare no conflict of interest.

## Supporting information

As a service to our authors and readers, this journal provides supporting information supplied by the authors. Such materials are peer reviewed and may be re‐organized for online delivery, but are not copy‐edited or typeset. Technical support issues arising from supporting information (other than missing files) should be addressed to the authors.

Supplementary
