# Supplementary material for: Side‐on Coordination in Isostructural Nitrous Oxide and Carbon Dioxide Complexes of Nickel
Source: Angew Chem Int Ed Engl. 2021 Feb 17;60(13):7077–81. doi: 10.1002/anie.202011301 (PMC8048599; doi:10.1002/anie.202011301)
Supplement: Supplementary file 1 — Supplementary [file ANIE-60-7077-s001.pdf]

Angewandte Chemie
Eine Zeitschrift der Gesellschaft Deutscher Chemiker

Supporting Information

Side-on Coordination in Isostructural Nitrous Oxide and Carbon Dioxide Complexes of Nickel

Braulio M. Puerta Lombardi[+], Chris Gendy[+], Benjamin S. Gelfand, Guy M. Bernard, Roderick E. Wasylishen, Heikki M. Tuononen, and Roland Roesler**

anie_202011301_sm_miscellaneous_information.pdf

Supporting Information

Table of Contents

General Considerations........4

Experimental Procedures........5

Synthesis of dimethylsilylbis(methylimidazolium) diiodide, $[(1)H_2]I_2$........5

Scheme S1. Synthesis of ligand **1**........5

Synthesis of dimethylsilylbis(methylimidazolium) bis(tetraphenylborate) $[(1)H_2][BPh_4]_2$........5

Synthesis of dimethylsilylbis(methylimidazolylidene), 1........6

Synthesis of (1)Ni(η^2-cod), 2........7

Scheme S2. Synthesis of nickel complex **2**........7

Synthesis of (1)Ni(*N*,*N*'-η^2-N_2O), 3........8

Scheme S3. Synthesis of nickel complexes **3** and **5**........8

Synthesis of (2)Ni(η^2-CO_2), 5........8

Generation of N_2O-($^{15}N_2$)........9

Scheme S4. Postulated succession of reactions leading to the generation of N_2O........10

Figure S1. A) Setup for N_2O-($^{15}N_2$) generation. **B)** Loading alkaline solution into compartment d. **C)** N_2O-($^{15}N_2$) generation in progress........11

Figure S2. A) Chromatogram of the gaseous product collected in the balloon using natural abundance NH_4NO_3 and urea. **B)** Mass-spectrum corresponding to the peak eluting at 1.360 s in chromatogram A. **C)** Chromatogram of the gaseous product collected in the balloon using $^{15}NH_4{}^{15}NO_3$ and urea-($^{15}N_2$). **D)** Mass-spectrum corresponding to the peak eluting at 1.360 s in chromatogram C........12

Figure S3. A) Chromatogram of the reaction headspace after heating **3** (15 mg) in THF-d_8 (1 mL) at 70 °C for 1.5 h in a J. Young NMR tube. **B)** Mass-spectrum corresponding to the peak eluting at

1.360 s in chromatogram A. **C)** Mass-spectrum corresponding to the peak eluting at 2.554 s in chromatogram A. 13
Figure S4. ^{1}H NMR spectrum of crude [(**1**)H_2]I_2 (400 MHz, 298 K, CD_2Cl_2). 14
Figure S5. ^{1}H NMR spectrum of [(**1**)H_2][BPh_4]$_2$ in air (contains H_2O, see dry compound below) (400 MHz, 298 K, CD_3CN) 15
Figure S6. ^{1}H NMR spectrum of [(**1**)H_2][BPh_4]$_2$ (400 MHz, 298 K, CD_3CN). 16
Figure S7. ^{13}C DEPTQ NMR spectrum of [(**1**)H_2][BPh_4]$_2$ (101 MHz, 298 K, CD_3CN) 17
Figure S8. ^{1}H-^{13}C HSQC spectrum of [(**1**)H_2][BPh_4]$_2$ (400 MHz, 298 K, CD_3CN). 18
Figure S9. ^{1}H-^{13}C HMBC spectrum of [(**1**)H_2][BPh_4]$_2$ (400 MHz, 298 K, CD_3CN). 19
Figure S10. ^{1}H NMR spectrum of **1** (400 MHz, 298 K, C_6D_6). 20
Figure S11. ^{13}C DEPTQ NMR spectrum of **1** (101 MHz, 298 K, C_6D_6). 21
Figure S12. ^{1}H-^{13}C HSQC spectrum of **1** (400 MHz, 298 K, C_6D_6). 22
Figure S13. ^{1}H-^{13}C HMBC spectrum of **1** (400 MHz, 298 K, C_6D_6). 23
Figure S14. ^{1}H NMR spectrum of **2** (400 MHz, 298 K, THF-d_8). 24
Figure S15. ^{1}H NMR spectrum of **2** (400 MHz, 195 K, THF-d_8). 25
Figure S16. ^{13}C DEPTQ NMR spectrum of **2** (101 MHz, 195 K, THF-d_8). 26
Figure S17. ^{1}H-^{13}C HSQC spectrum of **2** (400 MHz, 195 K, THF-d_8). 27
Figure S18. ^{1}H-^{13}C HMBC spectrum of **2** (400 MHz, 195 K, THF-d_8). 28
Figure S19. ^{13}C CP/MAS NMR spectrum of **3**, ν_{rot} = 10 kHz, acquired at 225 K at 11.75 T. 29
Figure S20. ^{15}N CP/MAS NMR spectrum of **3**, acquired at 225 K at 11.75 T with ν_{rot} = 10 kHz. 30
Figure S21. ^{13}C CP/MAS NMR spectrum of **5**, acquired at RT at 11.75 T with ν_{rot} = 14 kHz. 31
Figure S22. Baseline-corrected FT-IR for $^{14}N_2$-**3** at RT with KBr pellet. 32
Figure S23. Baseline-corrected FT-IR for isotopically-labelled $^{15}N_2$-**3** at RT with KBr pellet. 32
Figure S24. Superimposed inset of $^{15}N_2$-**3** (gray) and $^{14}N_2$-**3** (red). 33
Figure S25. Baseline-corrected FT-IR for **5** at RT with KBr pellet. 33
Figure S26. Overlaid FT-IR spectra for **3** and **3**-($^{15}N_2$) (99% isotopically enriched) with spectral difference below. 34
Figure S27. Solid-state structure of [(**1**)H_2][BPh_4]$_2$ 35
Figure S28. Solid-state structure of **2** 35
Figure S29. Solid-state structure of one of the two independent molecules of **3** 36
Figure S30. Solid-state structure of **5** 36
Table S1. Summary of Crystallographic Data for Compounds [(**1**)H_2][BPh_4], **2**, **3**, and **5**. 37
Computational Details 38
Geometries and properties of complexes 3, 4 and 5. 38

Calculated ^{15}N chemical shifts of 3. 38

Figure S31. Relative Gibbs energies for **3**, **4** and (**1**)Ni(N_2)O, and the associated transition states connecting the three species. 39

Comparison of ligand properties of N_2O and CO_2. 40

Table S2. Gibbs Energies Calculated for the Complexes M-CO_2 (Figure S32) and M-N_2O (in a.u.), and for Ligand Exchange Reaction M-CO_2 + N_2O ⇌ M-N_2O + CO_2 (in kJ mol^{-1}) Using Different Metal Fragments M. 41

Figure S32. M-CO_2 complexes used for the computational study summarised in Table S2. 41

Figure S33. Frontier orbitals of CO_2 and N_2O (PBE1PBE/def2-TZVP, isosurface value ±0.03), and their associated energies (in a.u.). 42

Table S3. Summary of Results (in kJ mol^{-1}) from Energy Decomposition Analyses Conducted for **3** and **5**. 42

Solid-State NMR 43

Referencing nitrogen chemical shifts. 43

Referencing nitrogen chemical shifts in solids. 44

NMR of nitrous oxide. 45

Table S4. Experimental and Calculated Isotropic Chemical Shifts (ppm) for **3**. 46

Table S5. PBE1PBE (top) and BP86 (bottom) Calculated Nitrogen and Oxygen Magnetic Shielding Tensors (ppm) for N_2O and **3**.[a,b] 46

Figure S34 A) Absolute ^{15}N magnetic shielding values for NH_3 and CH_3NO_2. **B)** Conversion of shielding values to chemical shifts relative to NH_3(l) using the equation δ = + 244.6 47

References 48

General Considerations

The synthesis and handling of compounds was performed under an argon atmosphere using an MBRAUN glove box or a double-manifold Schlenk line operating with argon, except for the protocol for $[(\mathbf{1})H_2]I_2$, which was conducted in the air. Pentane was dried using an MBRAUN solvent purification system and stored in a 500 mL glass vessel containing sodium. Benzene, toluene, *o*-xylene, and tetrahydrofuran were dried over potassium, distilled for storage into 500 mL air-tight vessels containing sodium/benzophenone ketyl, and vacuum-transferred into the reaction vessel. Nuclear magnetic resonance (NMR) spectra of samples in solution were acquired on Bruker Avance and Avance III 400 MHz spectrometers at 298 K, unless otherwise noted. ^{1}H and ^{13}C NMR chemical shifts were referenced to residual solvent peaks and naturally abundant ^{13}C resonances for all deuterated solvents: CH_2Cl_2-d_1 (5.32 ppm, ^{1}H) and CH_2Cl_2-d_2 (54.00 ppm, ^{13}C); acetonitrile-d_2 (1.94 ppm, ^{1}H) and acetonitrile-d_3 (1.32 ppm, ^{13}C); benzene-d_5 (7.16 ppm, ^{1}H) and benzene-d_6 (128.06 ppm, ^{13}C); tetrahydrofuran-d_7 (3.58 ppm, ^{1}H) and tetrahydrofuran-d_8 (67.21 ppm, ^{13}C).[1] X-ray crystallographic analyses were performed on a Bruker SMART APEX II CCD diffractometer using suitable single crystals coated in Paratone 8277 oil (Exxon) and mounted on glass-fiber loops. Measurements were processed with the Apex III software suite. Full crystallographic details can be found in each independently uploaded crystallographic information file (.cif). N_2O and N_2O-($^{15}N_2$) reagents were analyzed by gas-chromatography-mass-spectrometry (GC-MS) with an Agilent 7890B instrument. Infrared spectra were measured on a Nicolet Avatar FT-IR spectrometer, as KBr pellets sandwiched between two outer KBr layers. All elemental analyses were collected on a Perkin-Elmer Model 2400 series II analyzer. N-(2,6-diisopropylphenyl)imidazole,[2] and bis(iodomethyl)dimethylsilane[3] were prepared according to literature procedures. Bis(chloromethyl)dimethylsilane (Gelest), nitrous oxide (Air Liquide), carbon dioxide (Praxair), ammonium nitrate ($^{15}N_2$) (Cambridge Isotope Laboratories), $^{15}N_2$-urea (Millipore-Sigma) and all other reagents (Millipore-Sigma, Alfa Aesar) were used as received.

Experimental Procedures

Synthesis of dimethylsilylbis(methylimidazolium) diiodide, [(1)H_2]I_2. In air, a 100 mL round-bottom flask was charged with bis(iodomethyl)dimethylsilane (4.10 g, 12.0 mmol), and 1-(2,6-diisopropylphenyl)imidazole (8.25 g, 36.1 mmol). Acetonitrile (70 mL) was then added and the solution was refluxed for 16 h. The reaction was allowed to cool to room temperature and volatiles were removed by rotary evaporation. The residue was triturated with toluene (30 mL) with the assistance of sonication (15 min), and the solvent was carefully decanted off the resulting thick residue. Further trituration with diethyl ether (3 × 20 mL) with sonication afforded an off-white solid of moderate purity according to NMR. This was filtered, dried in vacuo, and subjected to anion exchange to yield [(**1**)H_2][BPh_4]$_2$ without further purification.

^{1}H NMR (400 MHz, CD_2Cl_2) δ 0.54 (s, 6H, Si(C**H**$_3$)$_2$), 1.18 (d, $^3J_{HH}$ = 6.8 Hz, 12H, CH(C**H**$_3$)$_2$), 1.22 (d, $^3J_{HH}$ = 6.8 Hz, 12H, CH(C**H**$_3$)$_2$), 2.31 (sept, $^3J_{HH}$ = 6.8 Hz, 4H, C**H**(CH$_3$)$_2$), 4.99 (s, 4H, C**H**$_2$), 7.28 (vt, $^vJ_{HH}$ = 1.8 Hz, 2H, Im**H**), 7.34 (d, $^3J_{HH}$ = 7.9 Hz, 4H, *m*-C$_6$**H**$_3$), 7.57 (t, $^3J_{HH}$ = 7.9 Hz, 2H, *p*-C$_6$**H**$_3$), 8.41 (vt, $^vJ_{HH}$ = 1.8 Hz, 2H, Im**H**), 10.24 (s, 2H, N$_2$C**H**). ^{13}C NMR (101 MHz, CD_2Cl_2) δ –4.33 (s, Si(**C**H$_3$)$_2$), 24.2 (s, CH(**C**H$_3$)$_2$), 24.9 (s, CH(**C**H$_3$)$_2$), 29.1 (s, **C**H(CH$_3$)$_2$), 39.8 (s, **C**H$_2$), 125.0 (s, **C**$_2$N$_2$C), 125.1 (s, *m*-**C**$_6$H$_3$), 125.3 (s, **C**$_2$N$_2$C), 130.44 (s, *o*-**C**$_6$H$_3$), 132.3 (s, *p*-**C**$_6$H$_3$), 136.8 (s, N$_2$**C**H), 145.8 (s, *i*-**C**$_6$H$_3$).

Scheme S1. Synthesis of ligand **1**.

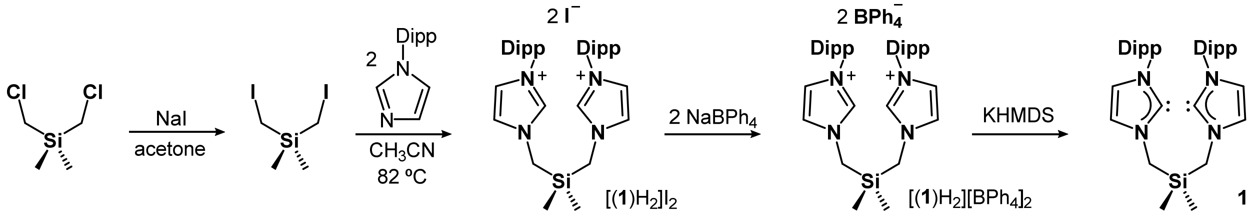

Synthesis of dimethylsilylbis(methylimidazolium) bis(tetraphenylborate) [(1)H_2][BPh_4]$_2$. Imidazolium salt [(**1**)H_2]I_2 (2.24 g, *ca.* 2.81 mmol) and sodium tetraphenylborate (1.93 g, 5.63 mmol) were charged to a 100 mL round-bottom flask. Anhydrous tetrahydrofuran (60 mL) was condensed over this mixture and the reaction was stirred at room temperature for 1 h. The reaction mixture was filtered through celite and the volatiles were removed in vacuo. The residue was triturated with dichloromethane (60 mL) and the white, solid product (2.59 g, 78%) was collected by filtration and washed with diethyl ether.

Solid $[(\mathbf{1})H_2][BPh_4]_2$ is hygroscopic and should be handled under an inert atmosphere. It decomposes in the presence of liquid water or alcohols, forming N-methyl-N'-(2,6-diisopropylphenyl)imidazolium tetraphenylborate among other, unidentified products. When exposed to air, $[(\mathbf{1})H_2][BPh_4]_2$ absorbs moisture but can be dehydrated via the following procedure: A 100 mL one-neck round-bottomed flask was charged with activated 4 Å molecular sieves (4.5 g) and hydrated $[(\mathbf{1})H_2][BPh_4]_2$ (2.081 g), and connected to a swivel-frit apparatus. THF (60 mL) was vacuum transferred to the flask and the mixture was stirred overnight. The sieves were subsequently filtered off, the flask with the filtrate was transferred onto another swivel frit, and the solvent was removed in vacuo. The remaining solid was suspended in pentane (40 mL), sonicated to a loose-powder consistency, and filtered. After thoroughly drying under vacuum, the product (1.68 g) was obtained as a white powder.

^{1}H NMR (400 MHz, CD_3CN) δ 0.29 (s, 6H, Si(C**H**$_3$)$_2$), 1.18 (d, $^3J_{HH}$ = 6.7 Hz, 12H, CH(C**H**$_3$)$_2$), 1.20 (d, $^3J_{HH}$ = 6.7 Hz, 12H, CH(C**H**$_3$)$_2$), 2.32 (sept, $^3J_{HH}$ = 6.7 Hz, 4H, C**H**(CH$_3$)$_2$), 4.31 (s, 4H, C**H**$_2$), 6.85 (t, $^3J_{HH}$ = 7.2 Hz, 8H, BPh_4^- p-C$_6$**H**$_5$), 7.00 (vt, $^3J_{HH}$ = 7.2 Hz, 16H, BPh_4^- o-C$_6$**H**$_5$), 7.30 (m, 16H, BPh_4^- m-C$_6$**H**$_5$), 7.43 (d, $^3J_{HH}$ = 8.0 Hz, 4H, *m*-C$_6$**H**$_3$), 7.52 (dd, $^3J_{HH}$ = 1.6, $^4J_{HH}$ = 7.2 Hz, Hz, 2H, ImH), 7.61 (t, $^3J_{HH}$ = 8.0 Hz, 2H, *p*-C$_6$**H**$_3$), 7.66 (dd, $^3J_{HH}$ = 1.6 Hz, $^4J_{HH}$ = 1.6 Hz, 2H, Im**H**), 9.11 (s, 2H, N$_2$C**H**). ^{13}C NMR (101 MHz, CD_3CN) δ -5.39 (s, Si(**C**H$_3$)$_2$), 24.2 (s, CH(**C**H$_3$)$_2$), 24.5 (s, CH(**C**H$_3$)$_2$), 29.4 (s, **C**H(CH$_3$)$_2$), 40.1 (s, **C**H$_2$), 122.8 (s, BPh_4^- *p*-**C**$_6$H$_5$), 125.2 (s, **C**$_2$N$_2$C), 125.6 (s, *m*-**C**$_6$H$_3$), 126.3 (s, **C**$_2$N$_2$C), 126.6 (q, $^2J_{CB}$ = 2.8 Hz, BPh_4^- *o*-**C**$_6$H$_5$), 131.1 (s, *o*-**C**$_6$H$_3$), 132.8 (s, *p*-**C**$_6$H$_3$), 136.7 (q, $^3J_{CB}$ = 1.4 Hz, BPh_4^- *m*-**C**$_6$H$_5$), 137.1 (s, N$_2$**C**H), 146.5 (s, *i*-**C**$_6$H$_3$), 164.8 (q, $^1J_{CB}$ = 49.3 Hz, BPh_4^- *i*-**C**$_6$H$_5$).

Synthesis of dimethylsilylbis(methylimidazolylidene), 1. (2.00 g, 1.69 mmol) and KHMDS (0.68 g, 3.38 mmol) were charged to a round-bottom flask. Toluene (50 mL) was vacuum transferred to the flask and the reaction was stirred at room temperature for 1 h. The solids were then filtered off over celite and the solvents were removed in vacuo. The residue was triturated twice with cold pentane, in which **1** is sparingly soluble, yielding an off-white solid (0.44 g, 48%). Further purification by crystallization from pentane is possible but generally not required. Solutions of **1** in hydrocarbon solvents decompose at room temperature over the course of several days, but no signs of decomposition were detected when the solid was stored over two months at –35 °C.

^1H NMR (400 MHz, C_6D_6) δ 0.41 (s, 6H, Si(C**H**$_3$)$_2$), 1.11 (d, $^3J_{HH}$ = 6.9 Hz, 12H, CH(C**H**$_3$)$_2$), 1.22 (d, $^3J_{HH}$ = 6.9 Hz, 12H, CH(C**H**$_3$)$_2$), 2.82 (sept, $^3J_{HH}$ = 6.9 Hz, 4H, C**H**(CH$_3$)$_2$), 3.81 (s, 4H, C**H**$_2$), 6.53 (s, 2H, Im**H**), 6.68 (s, 2H, Im**H**), 7.15 (d, $^3J_{HH}$ = 7.7 Hz, 4H, *m*-C$_6$**H**$_3$), 7.27 (t, $^3J_{HH}$ = 7.7 Hz, 2H, *p*-C$_6$**H**$_3$). ^{13}C NMR (101 MHz, C_6D_6) δ –3.36 (s, Si(**C**H$_3$)$_2$), 24.0 (s, CH(**C**H$_3$)$_2$), 24.7 (s, CH(**C**H$_3$)$_2$), 28.5 (s, **C**H(CH$_3$)$_2$), 40.9 (s, **C**H$_2$), 121.1 (s, **C**$_2$N$_2$C), 121.2 (s, **C**$_2$N$_2$C), 123.6 (s, *m*-**C**$_6$H$_3$), 128.8 (s, *p*-**C**$_6$H$_3$), 139.2 (s, *i*-**C**$_6$H$_3$), 146.5 (s, *o*-**C**$_6$H$_3$), 215.9 (s, N$_2$**C**). Anal. calcd. for $C_{34}H_{48}N_4Si$: C 75.50; H 8.95; N 10.36. Found: C 75.22; H 9.09; N 9.97.

Synthesis of (1)Ni(η^2-cod), 2. Ligand **1** (0.34 g, 0.62 mmol) and Ni(cod)$_2$ (0.17 g, 0.62 mmol) were charged to a 50 mL round-bottom flask attached to a pressure-equalizing swivel frit. Benzene (25 mL) was vacuum transferred to the flask and the mixture was stirred at room temperature for 2 h. The solvent was removed in vacuo and the residue was washed with cold pentane (–78 °C). The product was isolated by filtration as a yellow powder (0.31, 71%). Crystals of **2** suitable for X-ray crystallographic analysis were obtained by slow evaporation of a pentane solution.

Scheme S2. Synthesis of nickel complex **2**.

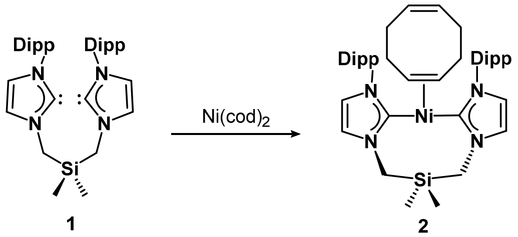

^1H NMR (400 MHz, 198 K, THF-*d*$_8$) δ (Selected resonances) 0.55 (bs, CH(C**H**$_3$)$_2$), 0.73 (bs, CH(C**H**$_3$)$_2$), 1.00 (bs, CH(C**H**$_3$)$_2$), 1.05 (bs, CH(C**H**$_3$)$_2$), 1.13 (bs, CH(C**H**$_3$)$_2$), 1.23 (bs, CH(C**H**$_3$)$_2$), 2.34 (bs, C**H**(CH$_3$)$_2$), 2.69 (bs, C**H**(CH$_3$)$_2$), 2.96 (d, $^2J_{HH}$ = 15.1 Hz, 1H, SiC**H$_2$**), 3.11 (bs, C**H**(CH$_3$)$_2$), 3.19 (d, $^2J_{HH}$ = 15.1 Hz, 1H, SiC**H$_2$**), 3.30 (bs, C**H**(CH$_3$)$_2$), 3.55 (d, $^2J_{HH}$ = 14.4 Hz, 1H, SiC**H$_2$**), 4.28 (s, 2H, COD-C**H**-Ni), 4.65 (d, $^2J_{HH}$ = 14.4 Hz, 1H, SiC**H$_2$**), 5.32 (m, COD-C**H**). ^{13}C NMR (101 MHz, 198 K, THF-*d*$_8$) δ (Selected resonances) 21.7 (s, CH(**C**H$_3$)$_2$), 22.6 (s, CH(**C**H$_3$)$_2$), 23.8 (s, CH(**C**H$_3$)$_2$), 24.0 (s, CH(**C**H$_3$)$_2$), 26.0 (s, CH(**C**H$_3$)$_2$), 26.3 (s, CH(**C**H$_3$)$_2$), 26.5 (s, CH(**C**H$_3$)$_2$), 27.8 (s, CH(**C**H$_3$)$_2$), 28.5 (s, **C**H(CH$_3$)$_2$), 28.7 (s, **C**H(CH$_3$)$_2$), 29.0 (s, **C**H(CH$_3$)$_2$), 29.1 (s, **C**H(CH$_3$)$_2$), 31.5 (s, COD-**C**H$_2$), 33.1 (s, COD-**C**H$_2$), 33.7 (s, COD-**C**H$_2$), 34.0 (s, COD-**C**H$_2$), 38.7 (s, Si**C**H$_2$), 40.2 (s, Si**C**H$_2$), 90.0 (s, COD-**C**H-Ni), 200.4 (s, N$_2$**C**), 208.4 (s, N$_2$**C**). Anal. calcd. for $C_{42}H_{60}N_4SiNi$: C 71.28; H 8.55; N 7.92. Found: C 70.71; H 9.00; N 7.35.

Synthesis of (1)Ni(*N*,*N'*-η^2-N_2O), 3. Complex **2** (0.05 g, 0.07 mmol) was charged to a J. Young NMR tube and dissolved in *o*-xylene (1.5 mL). The reaction was attached to a high-vacuum line and thoroughly degassed. N_2O (1 atm) was back-filled into the tube at room temperature and the reaction was agitated briefly and allowed to stand. The product crystallized after 10 min. The supernatant was decanted and the crystals were washed with pentane (2 × 10 mL) and allowed to dry in an argon glovebox, without exposure to vacuum. The reaction was carried out three times and the combined product from three reactions was collected as bright yellow crystals (0.09 g in total, 66%). X-ray quality crystals were obtained directly from the J. Young tube, before washing with pentane. The product was insoluble in benzene, toluene, o-xylene, THF, and pentane. It dissolved in toluene and THF upon quantitative decomposition accompanied by evolution of N_2 (See Fig. S3) when heated to 70 °C under an inert atmosphere.

Scheme S3. Synthesis of nickel complexes **3** and **5**.

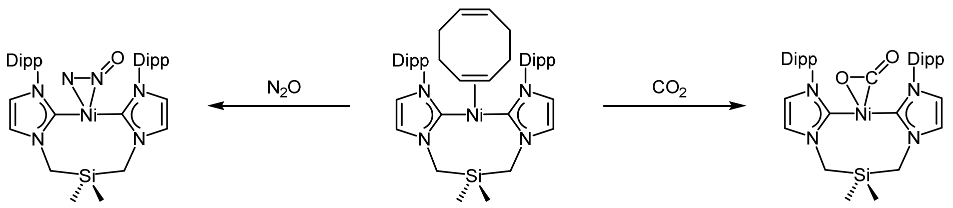

^{13}C CP/MAS NMR (225 K, ν_{rot} = 10 kHz) δ (Selected resonances) –5.74 (s, Si(**C**$H_3)_2$), –4.07 (s, Si(**C**$H_3)_2$), 19.4 (s, CH(**C**$H_3)_2$), 183.7 (s, N_2**C**), 192.5 (s, N_2**C**). ^{15}N CP/MAS NMR (225 K, ν_{rot} = 10 kHz) δ 187.5–194.0 (C_2**N_2**C), 311.8 (η^2-**N**NO), 364.7 (η^2-N**N**O). Anal. calcd. for $C_{34}H_{48}N_6SiONi$·(*o*-xylene)$_{0.5}$: C 65.51; H 7.67; N 12.07. Found: C 64.79; H 7.95; N 11.16.

Synthesis of (2)Ni(η^2-CO_2), 5. Complex **2** (0.18 g, 0.26 mmol) was charged to a round-bottom flask and attached to a pressure-equalizing swivel frit. Tetrahydrofuran (15 mL) was condensed into the flask and CO_2 (1 atm) was admitted to the tube at room temperature. A yellow precipitate was observed after 30 min and the reaction was stirred for an additional 30 min. The solid was filtered, washed with pentane (5 mL), and dried under vacuum, yielding **5** as a yellow microcrystalline solid (0.12 g, 73%). Single crystal X-ray crystallography and elemental analysis confirmed the presence of one equivalent of co-crystallized THF. The product was insoluble in benzene, toluene, o-xylene, THF, and pentane.

^{13}C CP/MAS NMR (298 K, ν_{rot} = 14 kHz) δ (Selected resonances) –5.16 (s, Si(**C**$H_3)_2$), –0.62 (s, Si(**C**$H_3)_2$), 167.3 (s, Ni**C**O_2), 188.2 (s, N_2**C**), 192.2 (s, N_2**C**). Anal. calcd. for $C_{35}H_{48}N_4SiO_2Ni\cdot(C_4H_8O)$: C 65.44; H 7.89; N 7.83. Found: C 64.44; H 7.90; N 7.26.

Generation of N_2O-($^{15}N_2$). We found that the industrial process for the manufacture of N_2O, discovered by Higgins, Berthollet, and Austin during the second half of the 16th century and based on the thermal decomposition of NH_4NO_3,[4] was ill-suited for the laboratory generation of isotopically labeled samples. Aside from the well-known explosion hazard associated with heating NH_4NO_3, in our hands this reaction was plagued by low yields, slow kinetics, and low product purity. Recent optimizations[5,6] and earlier, elaborate setups devised for this much investigated synthesis[7] indicate that others have encountered difficulties as well. Here we describe a simple, safe and reliable method for the generation of tens to hundreds of cm^3 $^{15}N_2O$ of satisfactory purity starting with labeled urea and a nitrate salt.

Solid pyrogallol, (0.25 g) was loaded via the top opening into section c of the custom-built bubbler shown in Figure S1A. The top of the vessel **b** was loaded with anhydrous $CaCl_2$ on top of a cotton wool plug, three-way valve **a** fitted with a balloon was attached to the top joint, section **d** side joint was capped, and the apparatus was evacuated. A 5 mL two-neck round-bottomed flask **e** equipped with a magnetic stir-bar was charged with $^{15}N_2$-urea (0.15 g, 2.42 mmol) and H_2SO_4 (0.4 mL) (note: exothermic) and placed under an N_2O atmosphere (natural abundance). The bubbler was then filled with N_2O and carefully loaded with 15% aqueous KOH solution in N_2O countercurrent, via the side joint, as depicted in Figure S1B, after which the two-neck round-bottom flask **e** was attached to the setup. In a separate vial, $^{15}NH_4{}^{15}NO_3$ (0.21 g, 2.50 mmol) was mixed with H_2SO_4 (0.4 mL) until a homogeneous solution was obtained. After the system was isolated from the N_2O source and opened to the balloon using three-way valve **a**, this solution was added to the urea-H_2SO_4 solution dropwise via syringe and the mixture was stirred at room-temperature until most of the alkaline pyrogallol in section **d** had been pushed to section **c**. The contents of the flask **e** were heated at 70 °C for 15 minutes on an oil bath, while $^{15}N_2$-N_2O was generated and collected in the balloon (Figure S1C). This was subsequently isolated from the bubbler using valve **a**, which was then detached from the apparatus and connected to a J. Young tube for $^{15}N_2$-N_2O addition. The collected gas was constituted primarily by N_2O/$^{15}N_2O$ in a 2:1 ratio, alongside trace amounts of CO_2 by-product (Figure S2).

Notes:

Prior to $^{15}N_2O$ generation, the reaction vessel can be filled with argon instead of N_2O, in order to collect a $^{15}N_2O$ – argon mixture. We found that the precipitation of **3** as a (**1**)Ni(*N*,*N*'-η^2-$^{15}N_2O$)-(**1**)Ni(*N*,*N*'-η^2-$^{14}N_2O$) mixture was more reliable with a $^{15}N_2O$-$^{14}N_2O$ mixture than the precipitation of pure (**1**)Ni(*N*,*N*'-η^2-$^{15}N_2O$) using $^{15}N_2O$ dilute with argon.

Rapid gas development in the flask **e** at the beginning of the reaction, in combination with the small diameter of the bubbling tube in **c** that is necessary for efficient CO_2 scrubbing, can lead to pressure build-up in the apparatus. A well-ventilated fume hood and a portable blast shield are strongly recommended.

$NH_4{}^{15}NO_3$ and $Na^{15}NO_3$ are expected to be just as suitable as $^{15}NH_4{}^{15}NO_3$ for $^{15}N_2O$ generation, but have not been tested in this regard.

Urea nitrate is formed upon addition of the in-situ prepared nitric acid solution to the reaction flask containing urea in H_2SO_4 (Scheme S4, equation 1).[8] Urea nitrate is quickly dehydrated by the H_2SO_4 reaction medium to form nitrourea (Scheme S4, equation 2)[9] which subsequently hydrolyzes to carbamic acid and the simplest nitramide, NH_2NO_2 (Scheme S4, equation 3). Carbamic acids are generally unstable and decompose to the parent amine (NH_3) and carbon dioxide (Scheme S4, equation 4),[10] while NH_2NO_2 decomposes to N_2O and water (Scheme S4, equation 5).[9,11] Evidence for N_2O evolution from the decomposition of organic nitramides has been well-documented.[12-15]

Scheme S4. Postulated succession of reactions leading to the generation of N_2O.

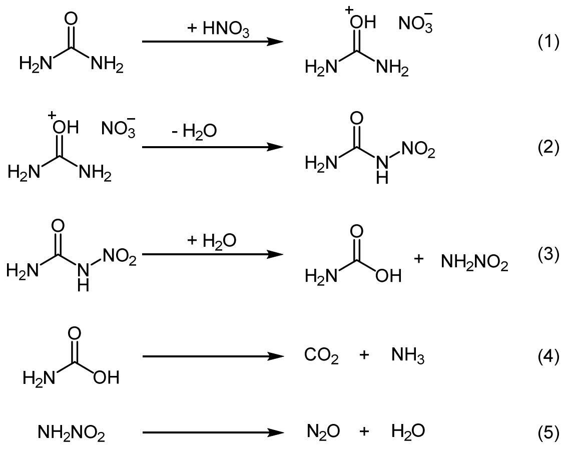

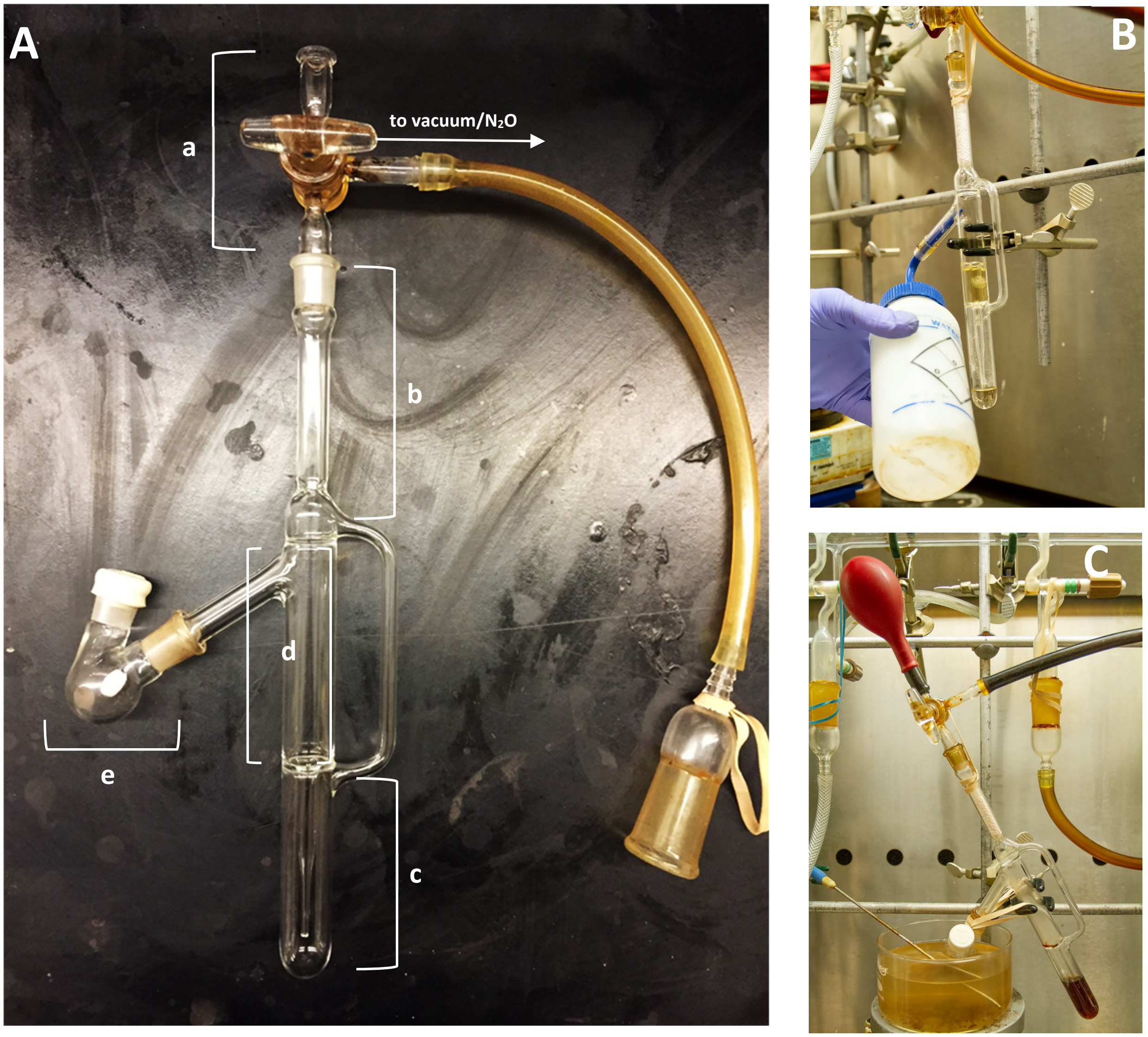

Figure S1. A) Setup for N_2O-($^{15}N_2$) generation. **B)** Loading alkaline solution into compartment d. **C)** N_2O-($^{15}N_2$) generation in progress.

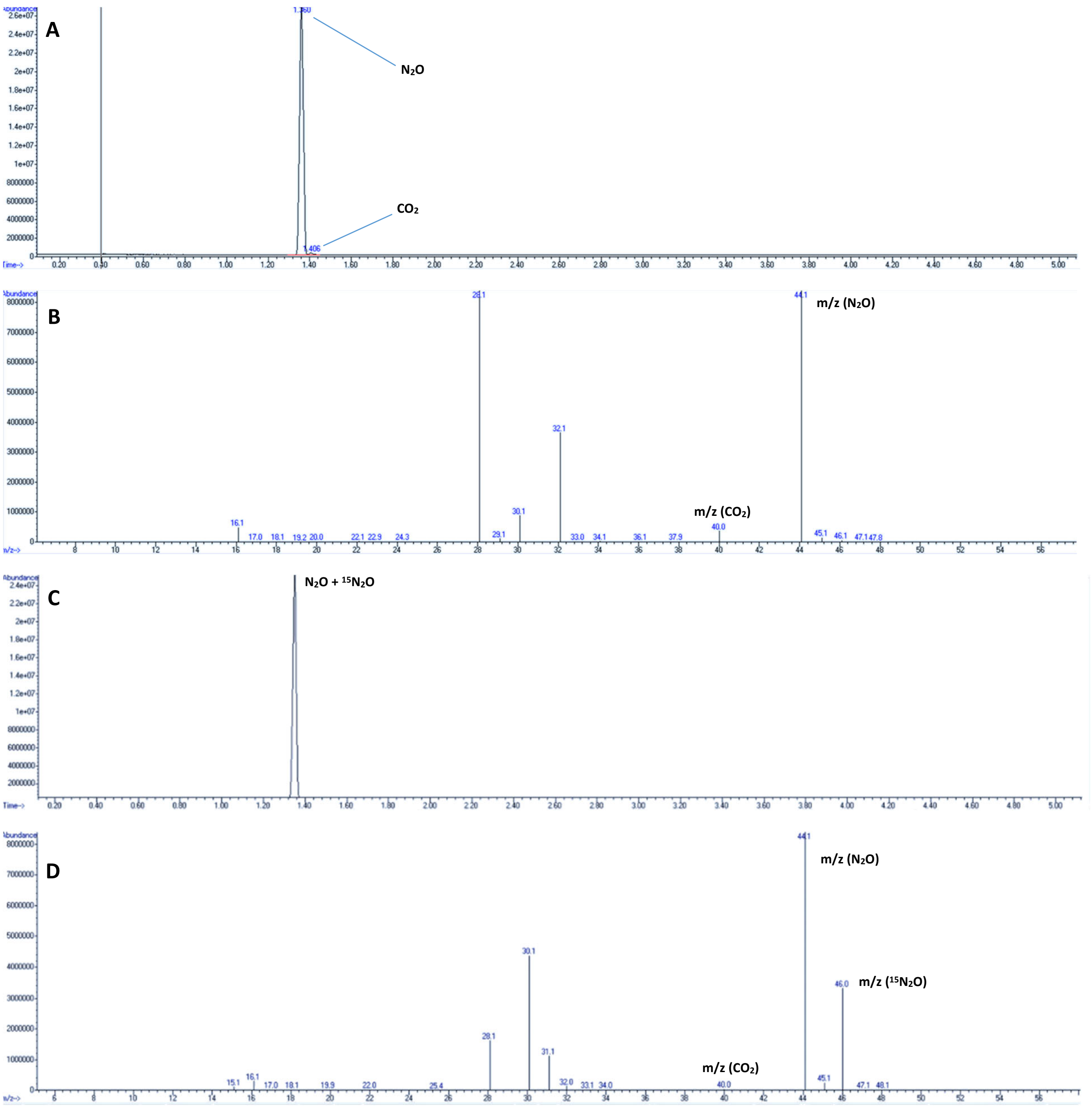

Figure S2. A) Chromatogram of the gaseous product collected in the balloon using natural abundance NH_4NO_3 and urea. **B)** Mass-spectrum corresponding to the peak eluting at 1.360 s in chromatogram A. **C)** Chromatogram of the gaseous product collected in the balloon using $^{15}NH_4{}^{15}NO_3$ and urea-($^{15}N_2$). **D)** Mass-spectrum corresponding to the peak eluting at 1.360 s in chromatogram C.

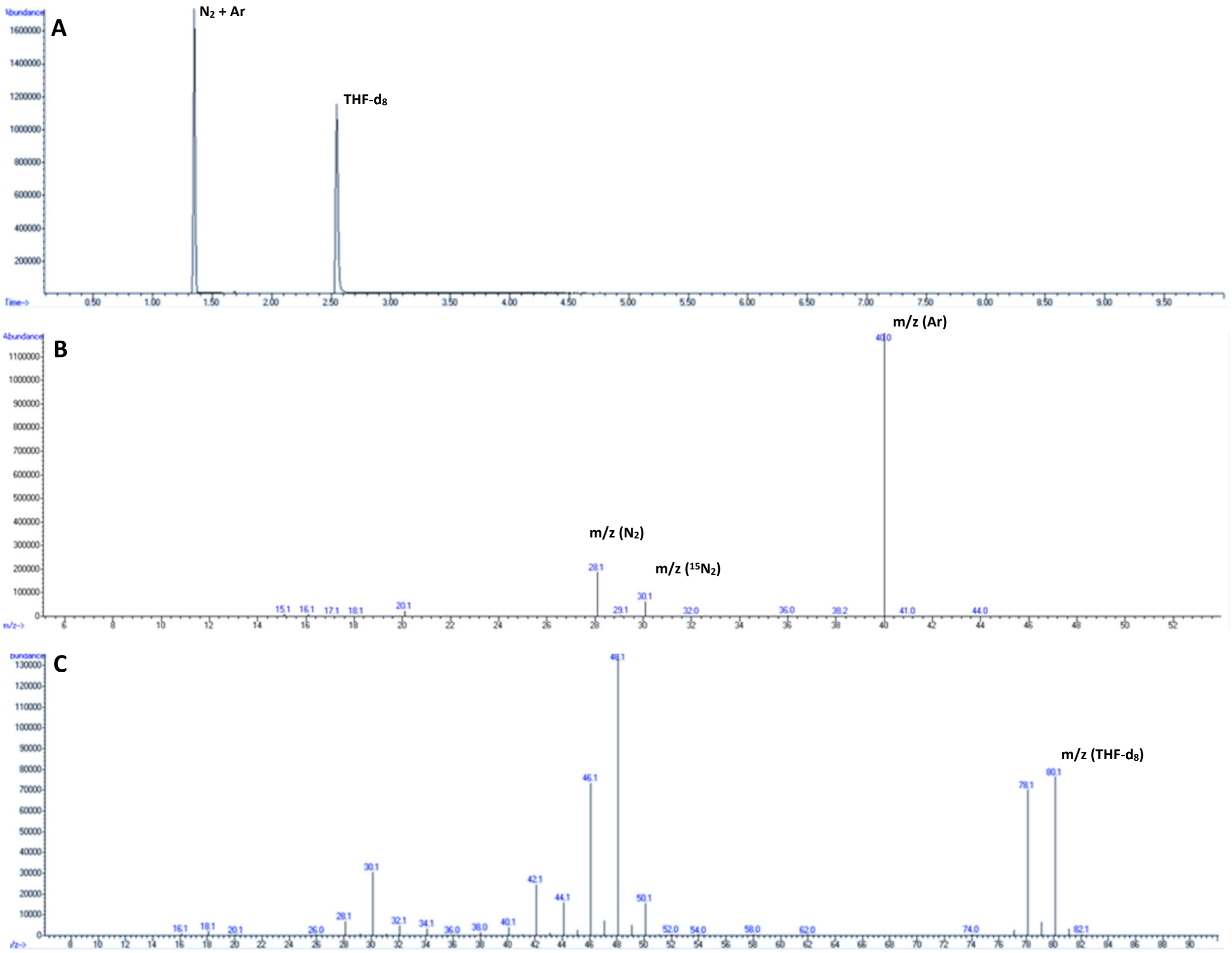

Figure S3. A) Chromatogram of the reaction headspace after heating **3** (15 mg) in THF-d_8 (1 mL) at 70 °C for 1.5 h in a J. Young NMR tube. **B)** Mass-spectrum corresponding to the peak eluting at 1.360 s in chromatogram A. **C)** Mass-spectrum corresponding to the peak eluting at 2.554 s in chromatogram A.

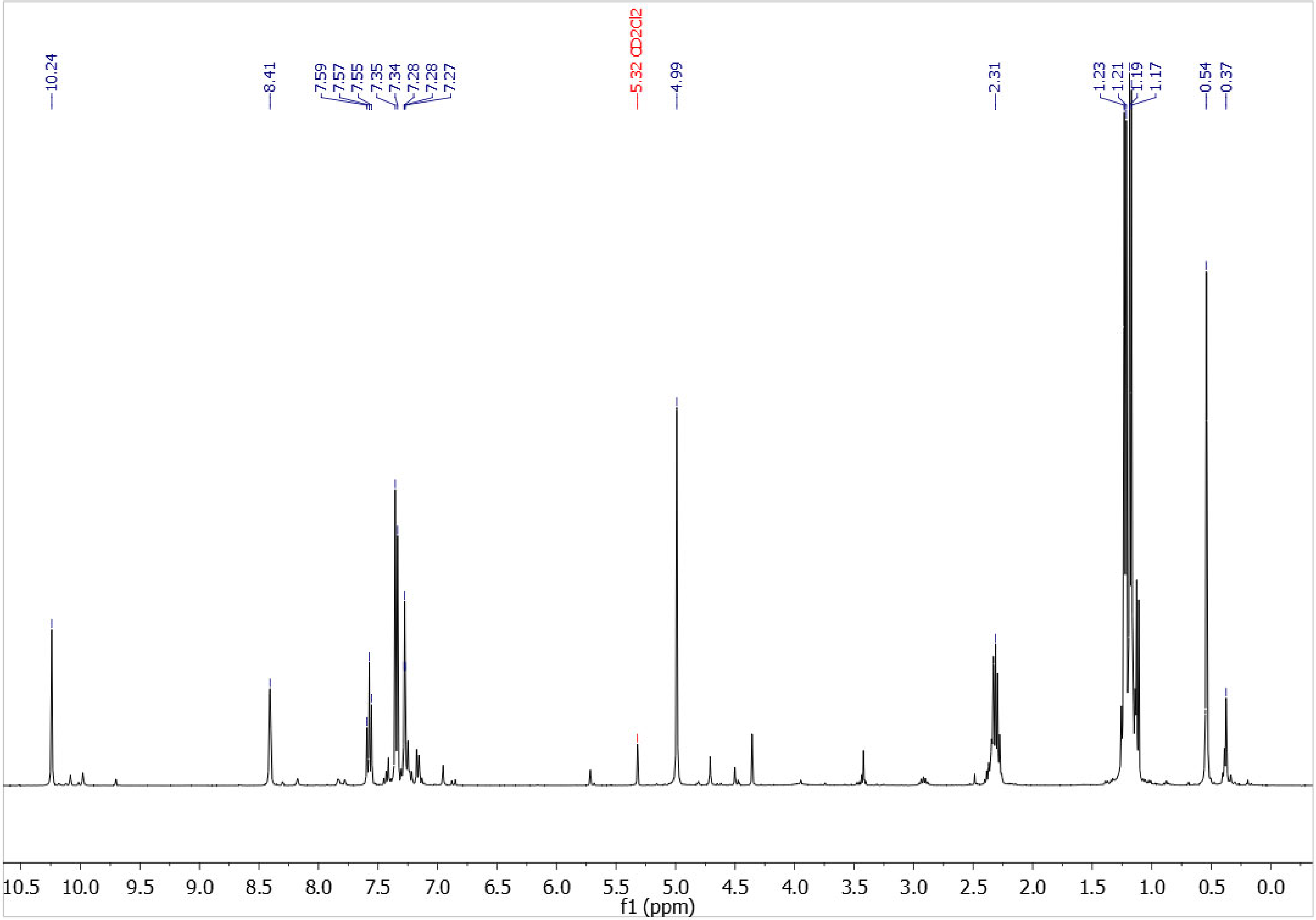

Figure S4. ^{1}H NMR spectrum of crude [(**1**)H_2]I_2 (400 MHz, 298 K, CD_2Cl_2).

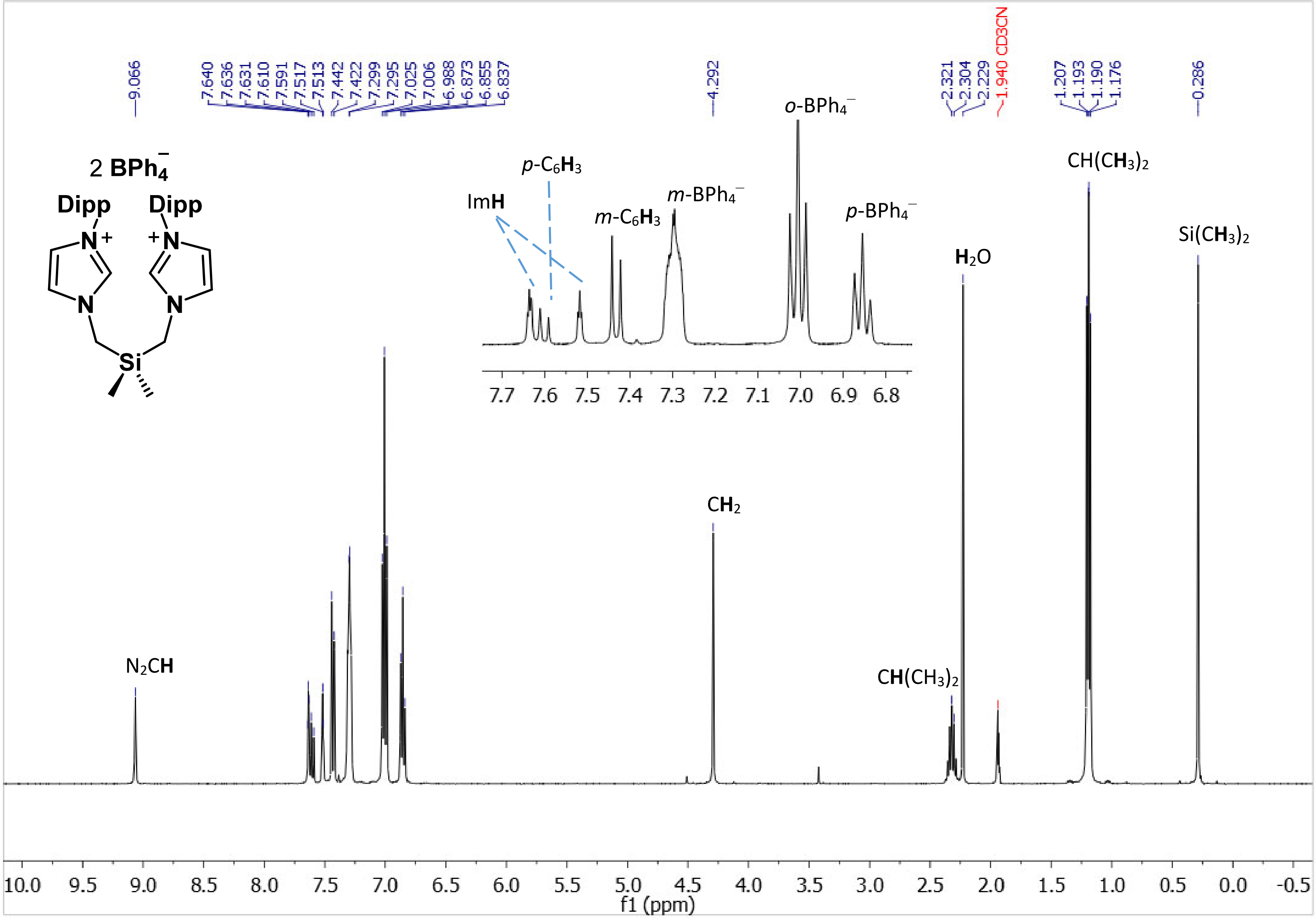

Figure S5. ¹H NMR spectrum of [(**1**)H_2][BPh_4]$_2$ in air (contains H_2O, see dry compound below) (400 MHz, 298 K, CD_3CN)

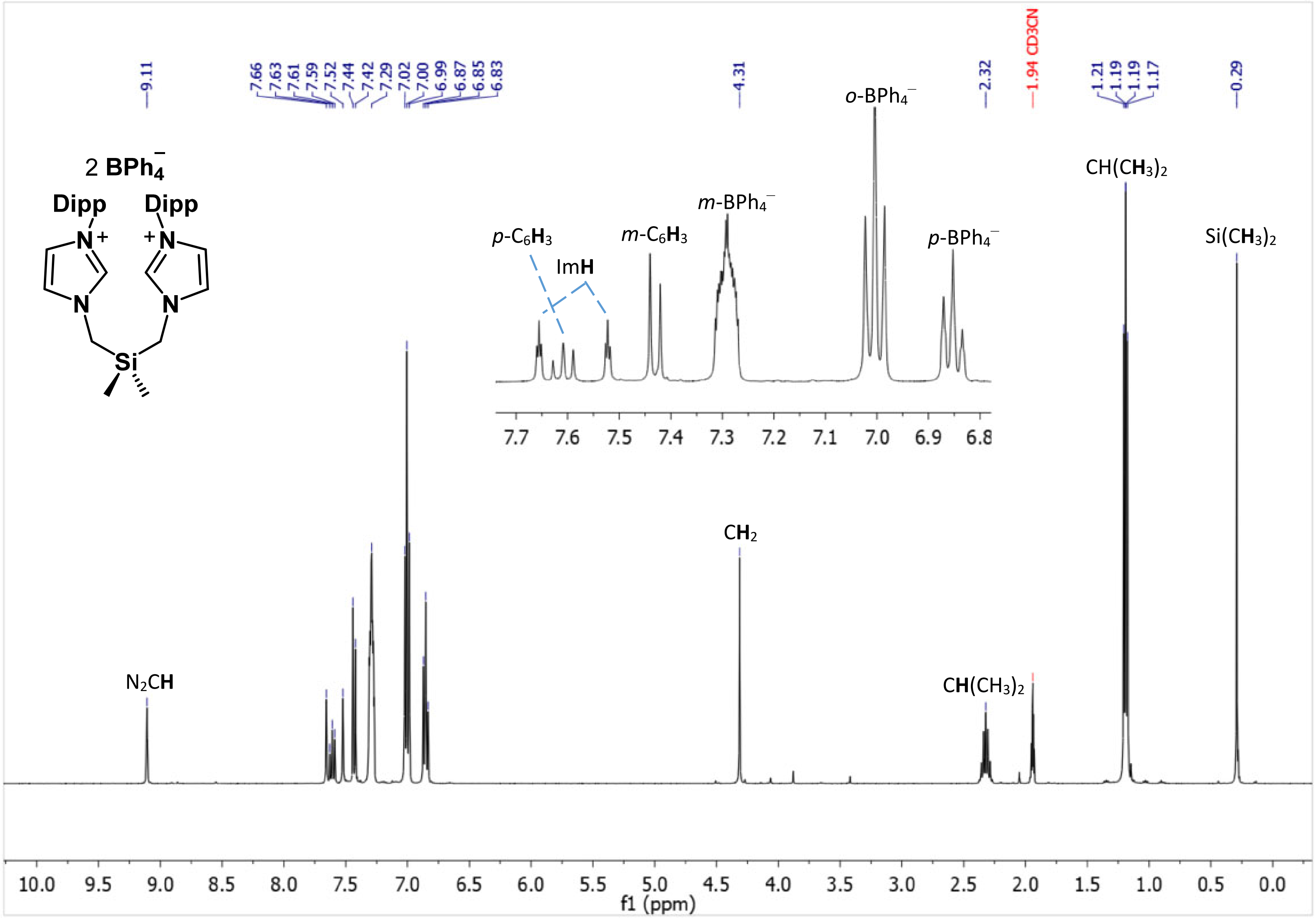

Figure S6. ^{1}H NMR spectrum of $[(\mathbf{1})H_2][BPh_4]_2$ (400 MHz, 298 K, CD_3CN).

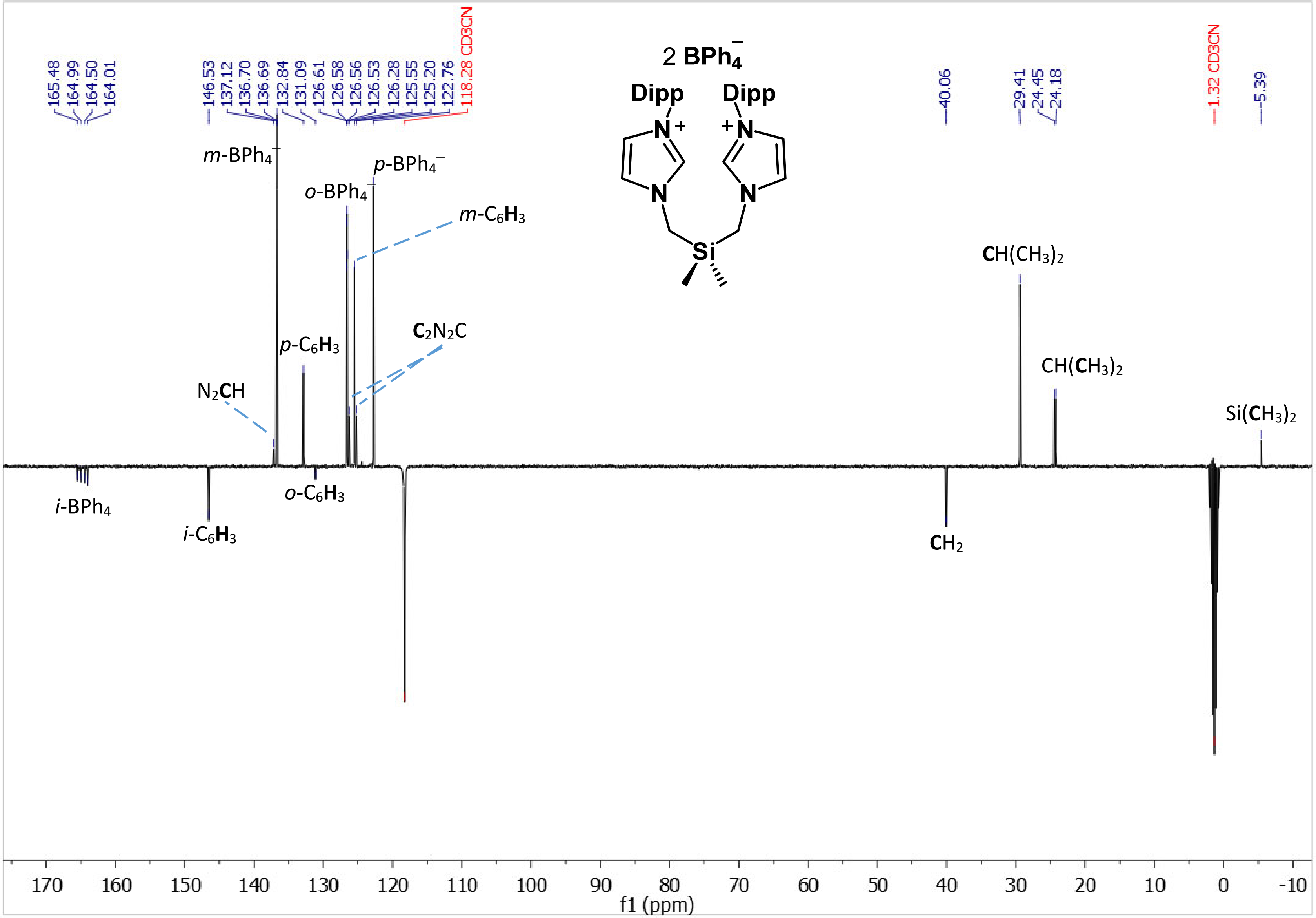

Figure S7. ^{13}C DEPTQ NMR spectrum of [(**1**)H_2][BPh_4]$_2$ (101 MHz, 298 K, CD_3CN).

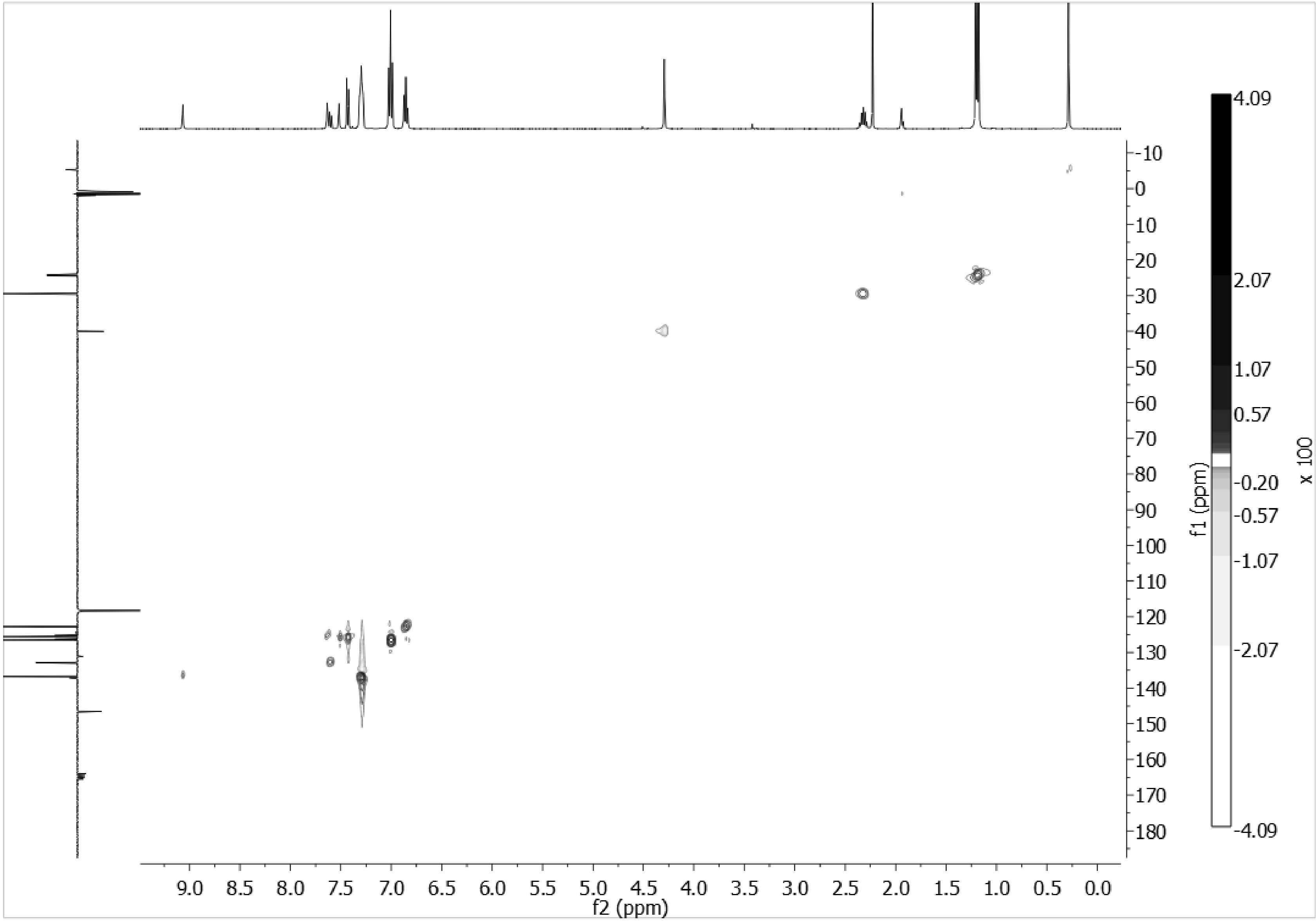

Figure S8. ^{1}H-^{13}C HSQC spectrum of $[(\mathbf{1})H_2][BPh_4]_2$ (400 MHz, 298 K, CD_3CN).

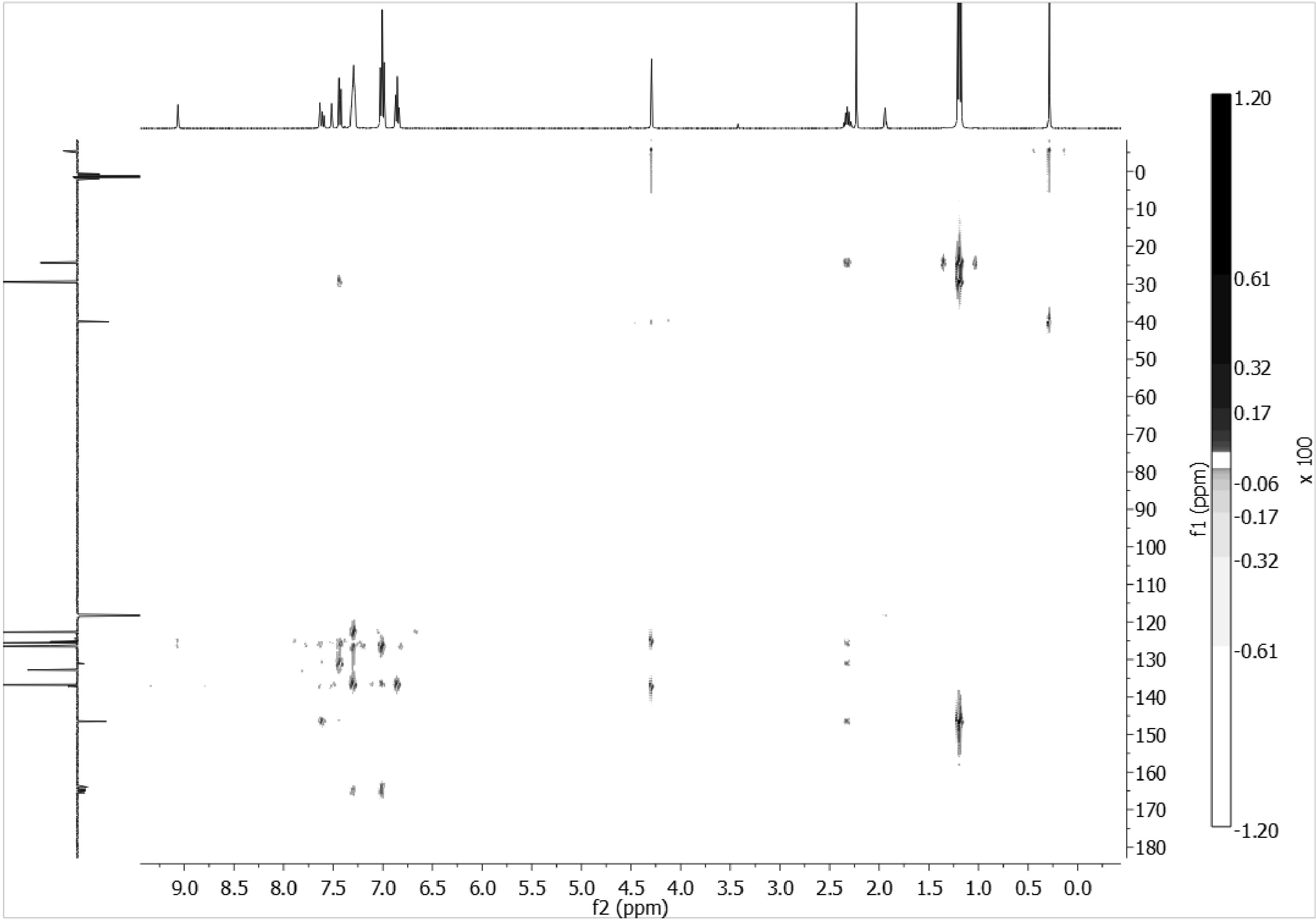

Figure S9. ^{1}H-^{13}C HMBC spectrum of $[(\mathbf{1})H_2][BPh_4]_2$ (400 MHz, 298 K, CD_3CN).

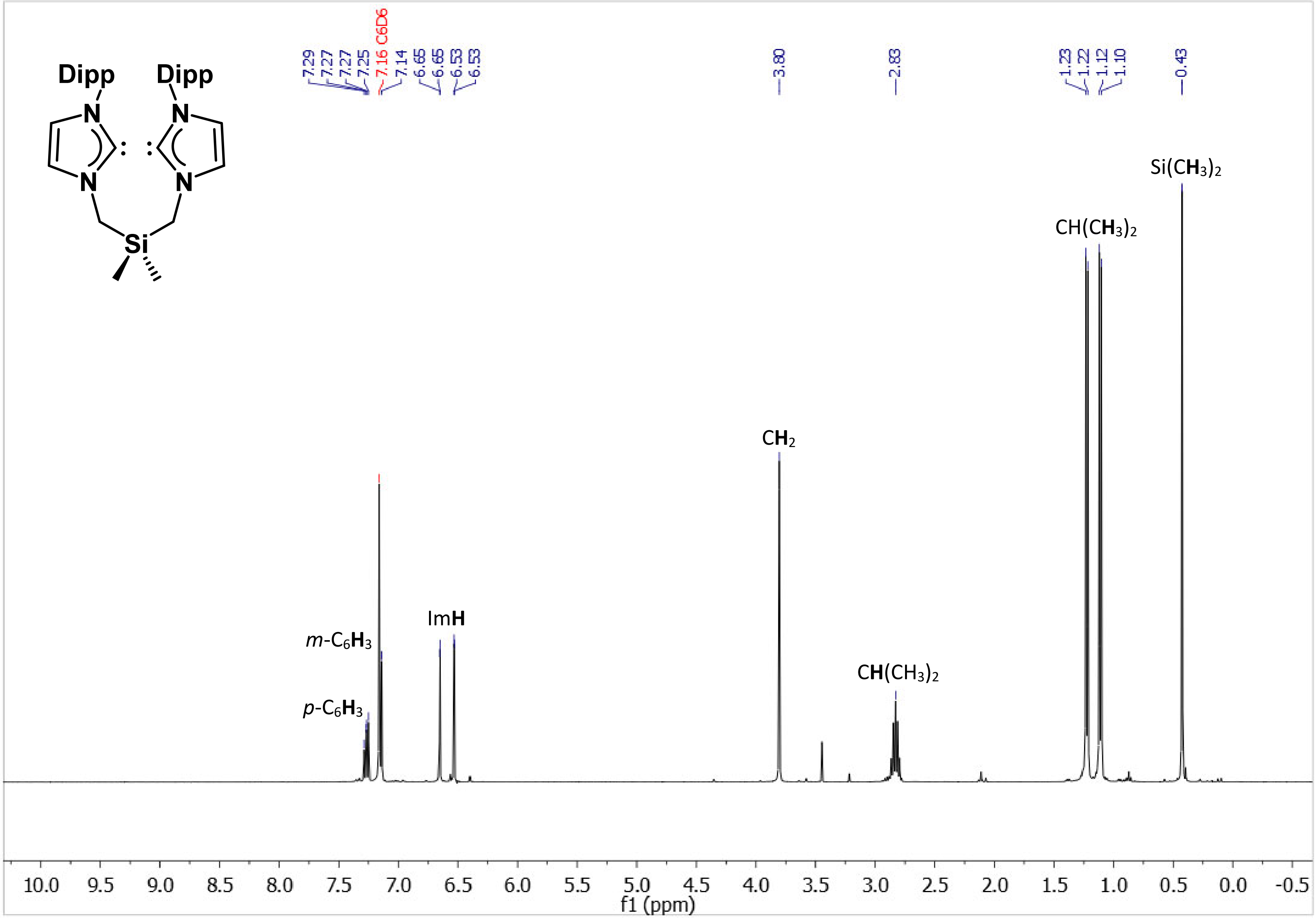

Figure S10. ^{1}H NMR spectrum of **1** (400 MHz, 298 K, C_6D_6).

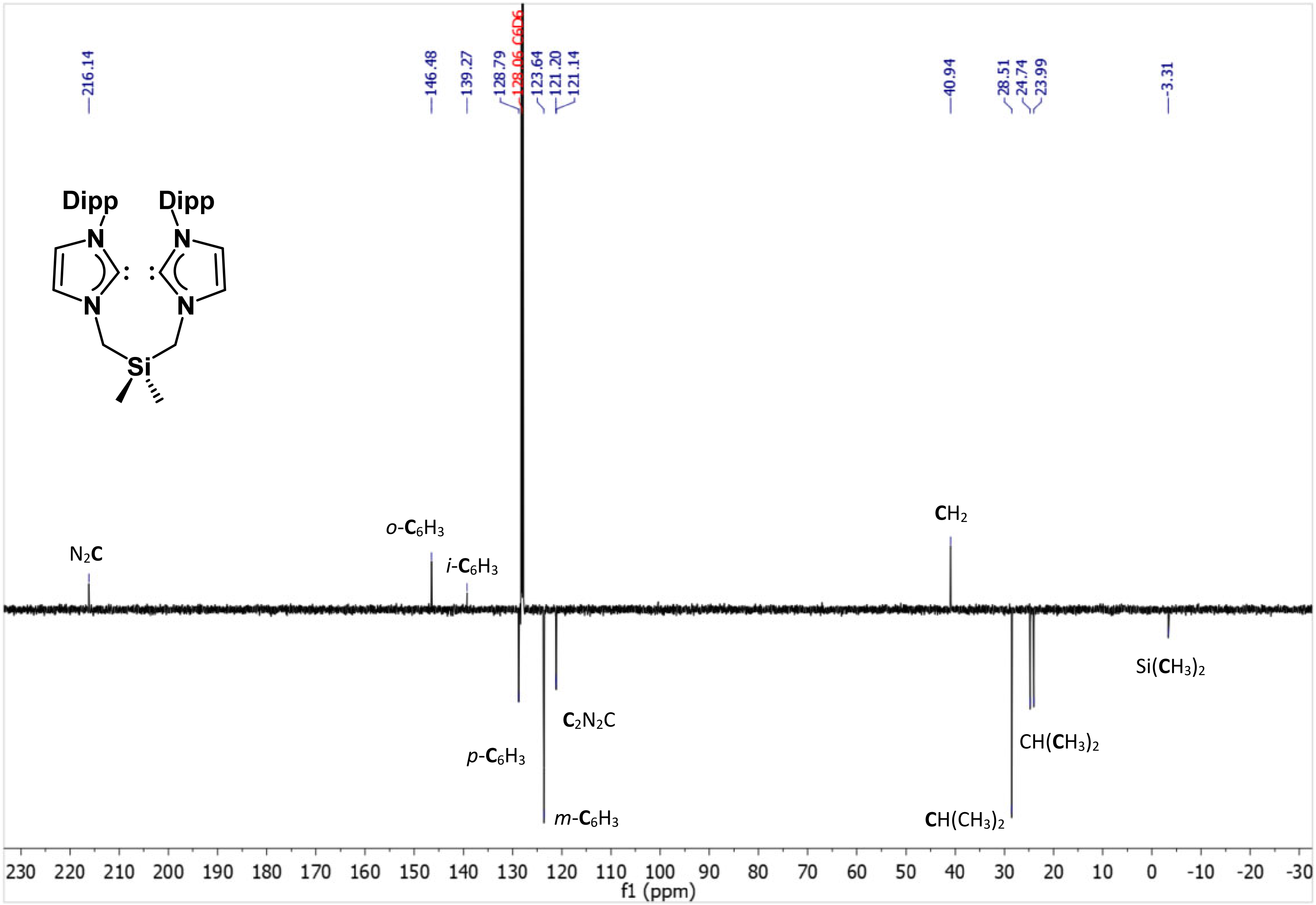

Figure S11. ^{13}C DEPTQ NMR spectrum of **1** (101 MHz, 298 K, C_6D_6).

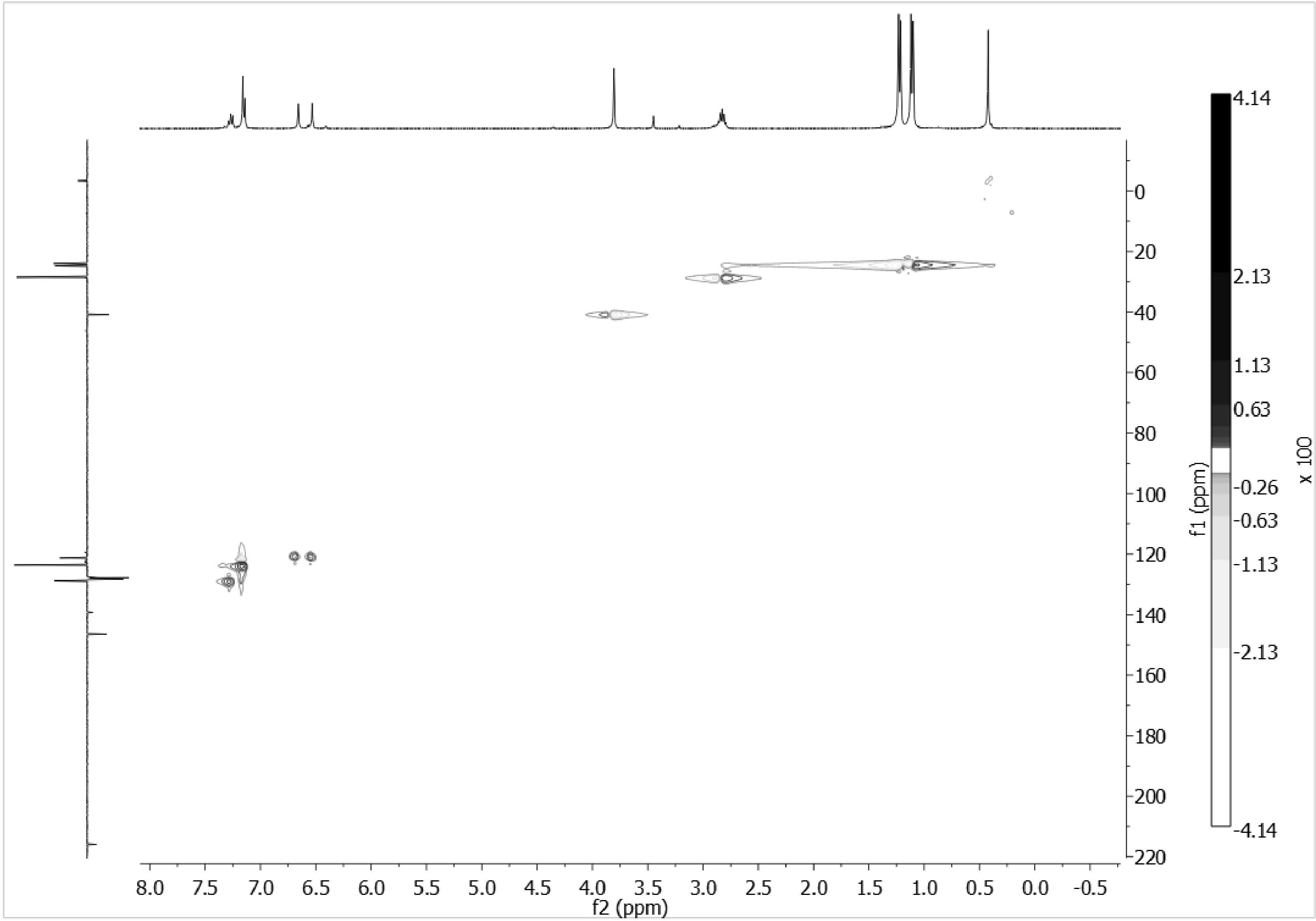

Figure S12. ^{1}H-^{13}C HSQC spectrum of **1** (400 MHz, 298 K, C_6D_6).

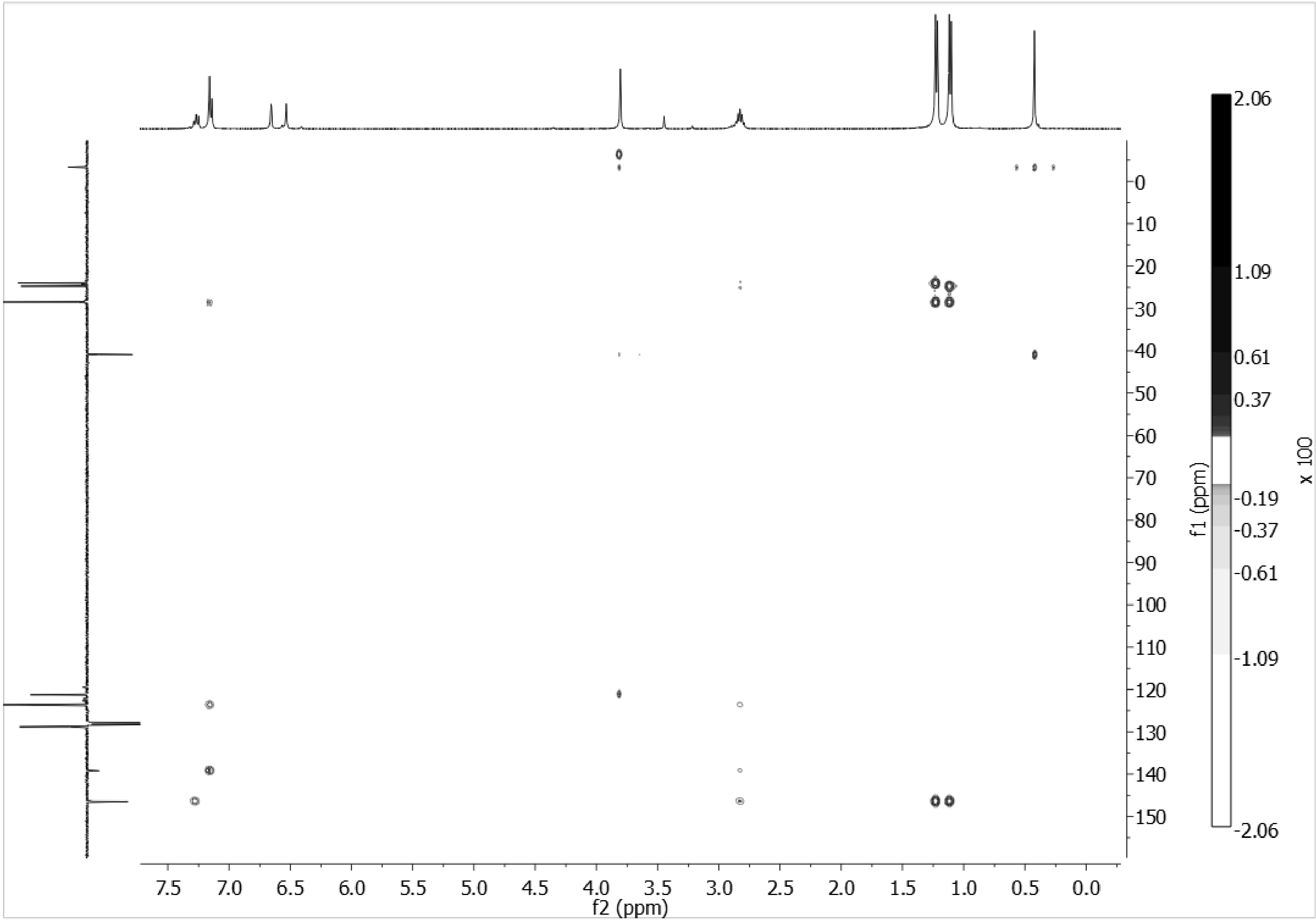

Figure S13. ^{1}H-^{13}C HMBC spectrum of **1** (400 MHz, 298 K, C_6D_6).

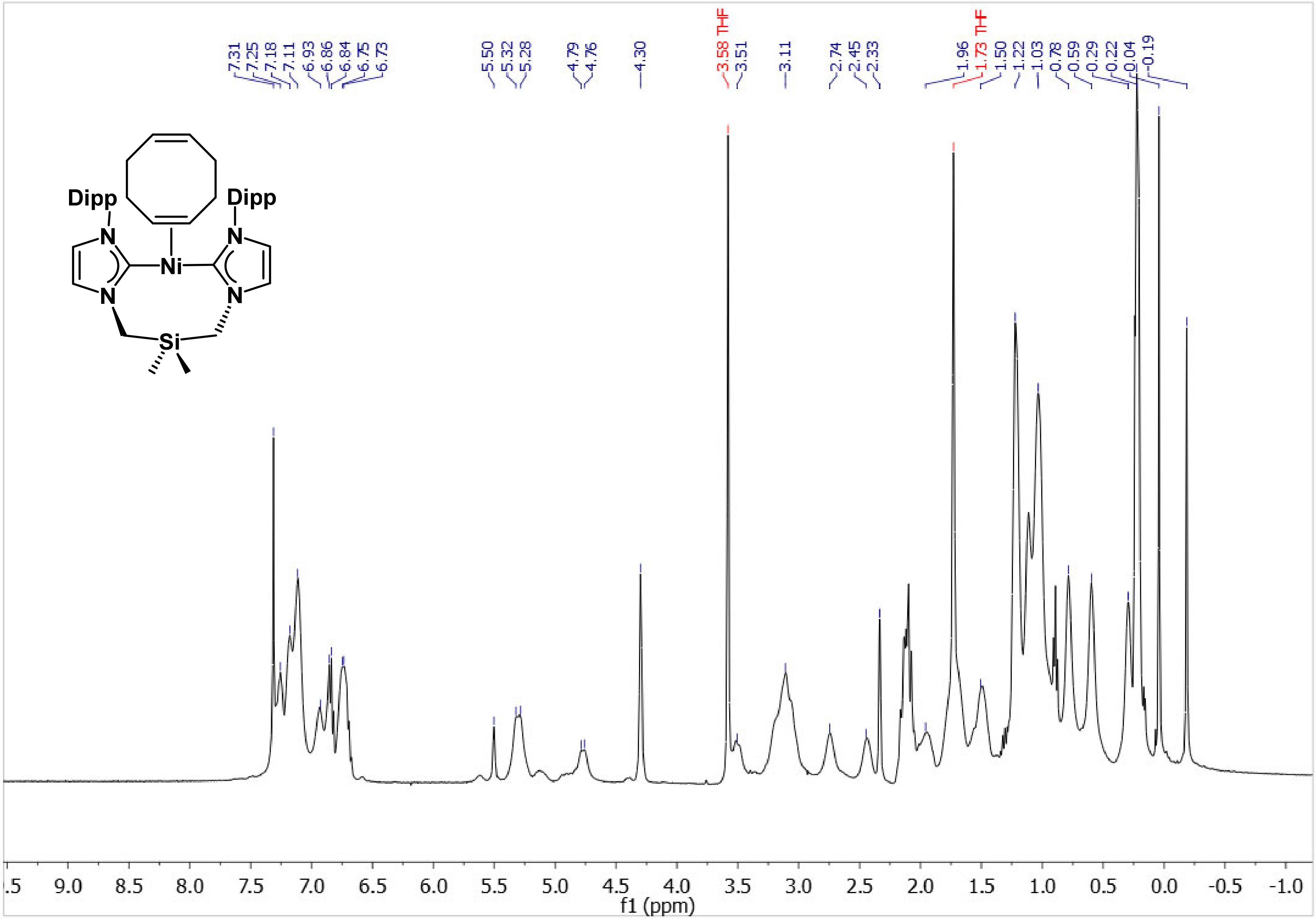

Figure S14. ^{1}H NMR spectrum of **2** (400 MHz, 298 K, THF-d_8).

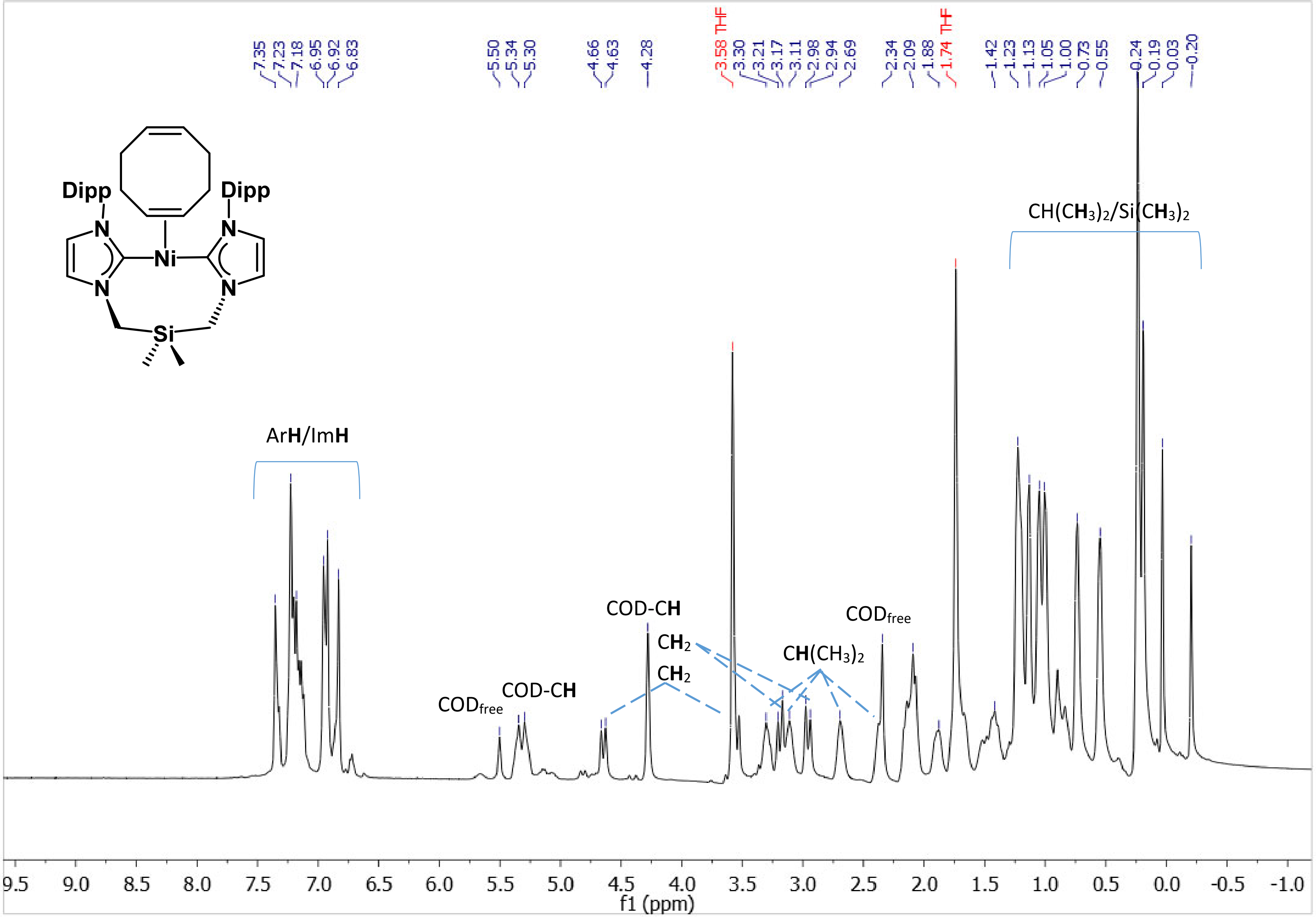

Figure S15. ^{1}H NMR spectrum of **2** (400 MHz, 195 K, THF-d_8).

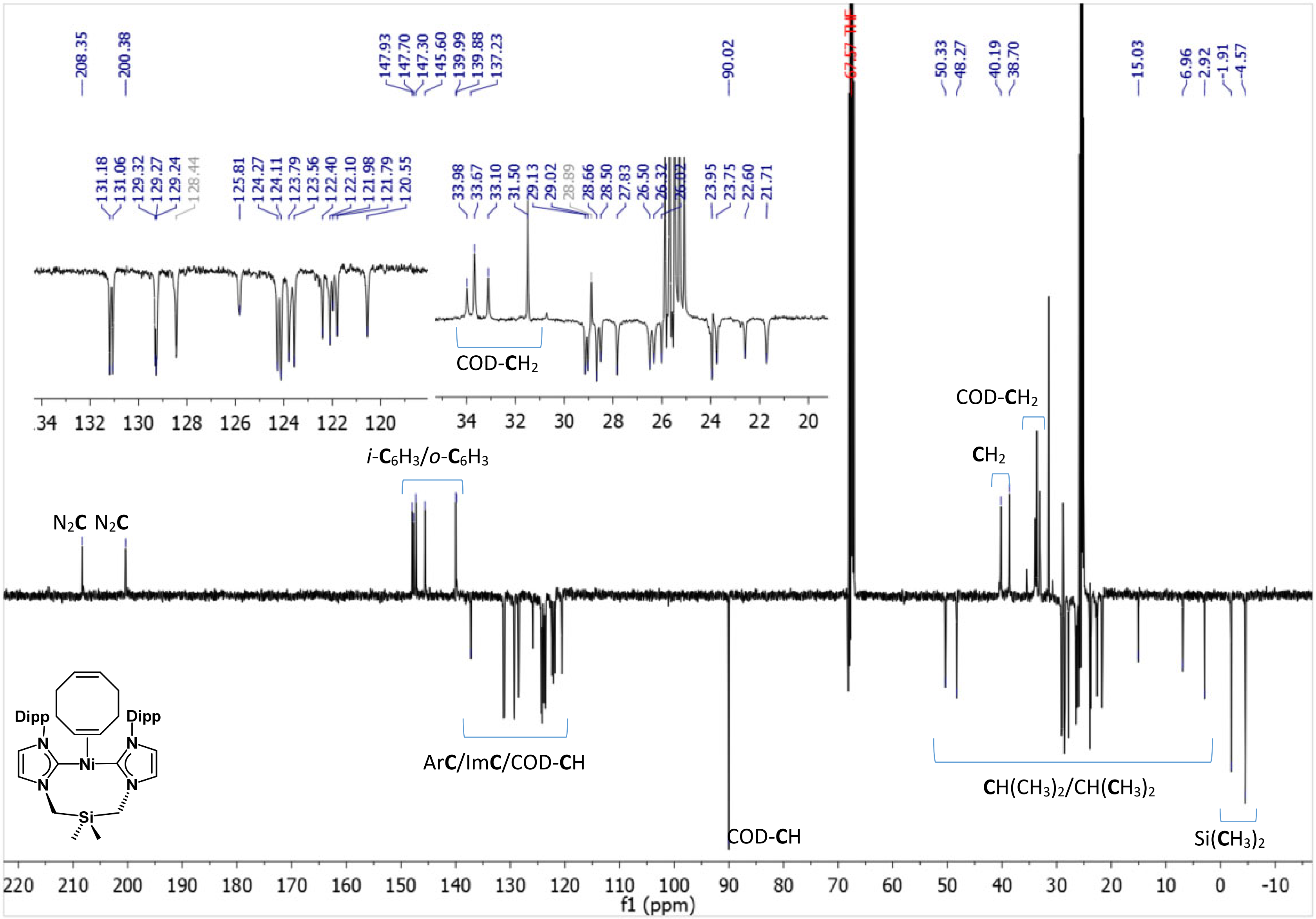

Figure S16. ^{13}C DEPTQ NMR spectrum of **2** (101 MHz, 195 K, THF-d_8).

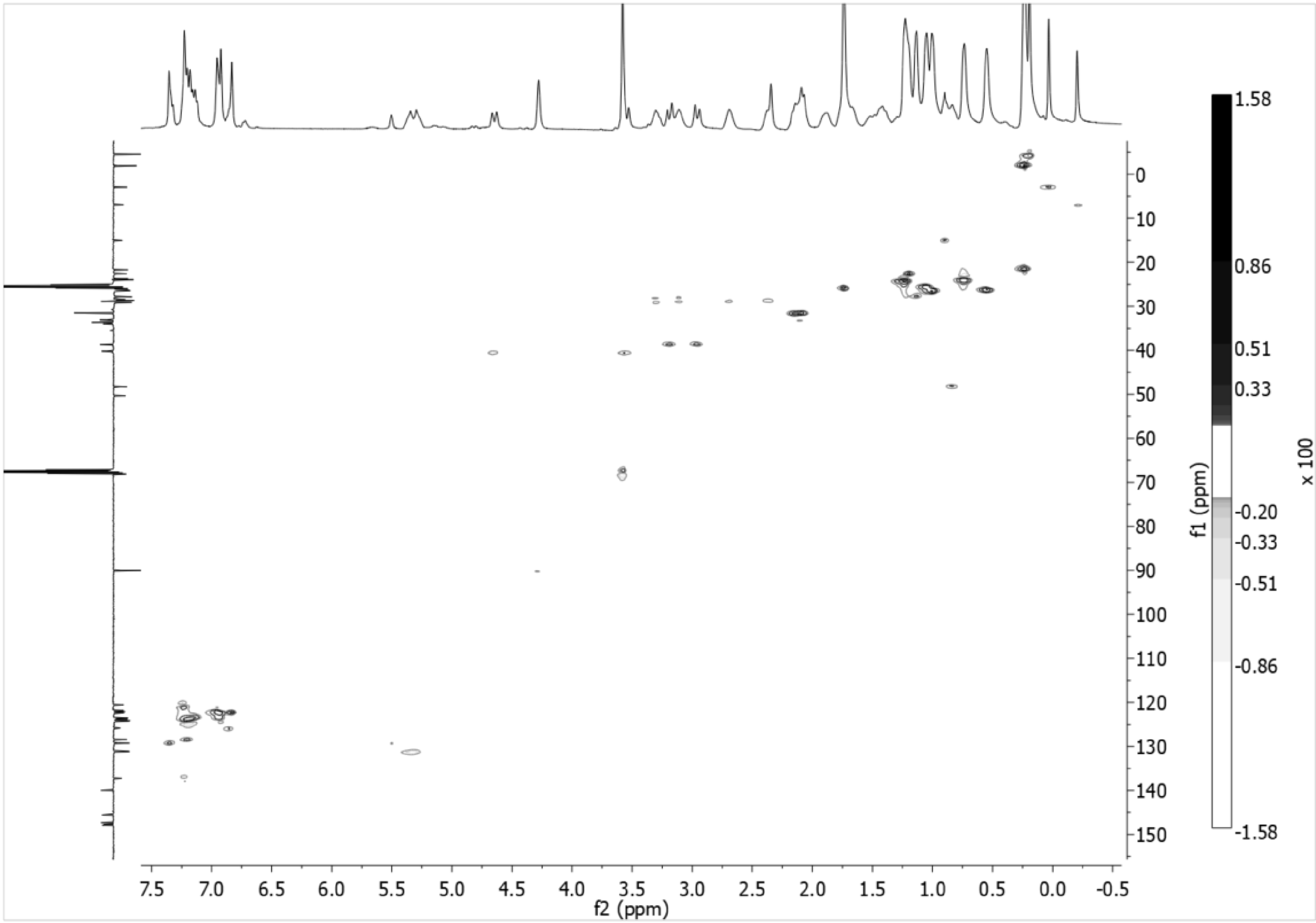

Figure S17. ^{1}H-^{13}C HSQC spectrum of **2** (400 MHz, 195 K, THF-d_8).

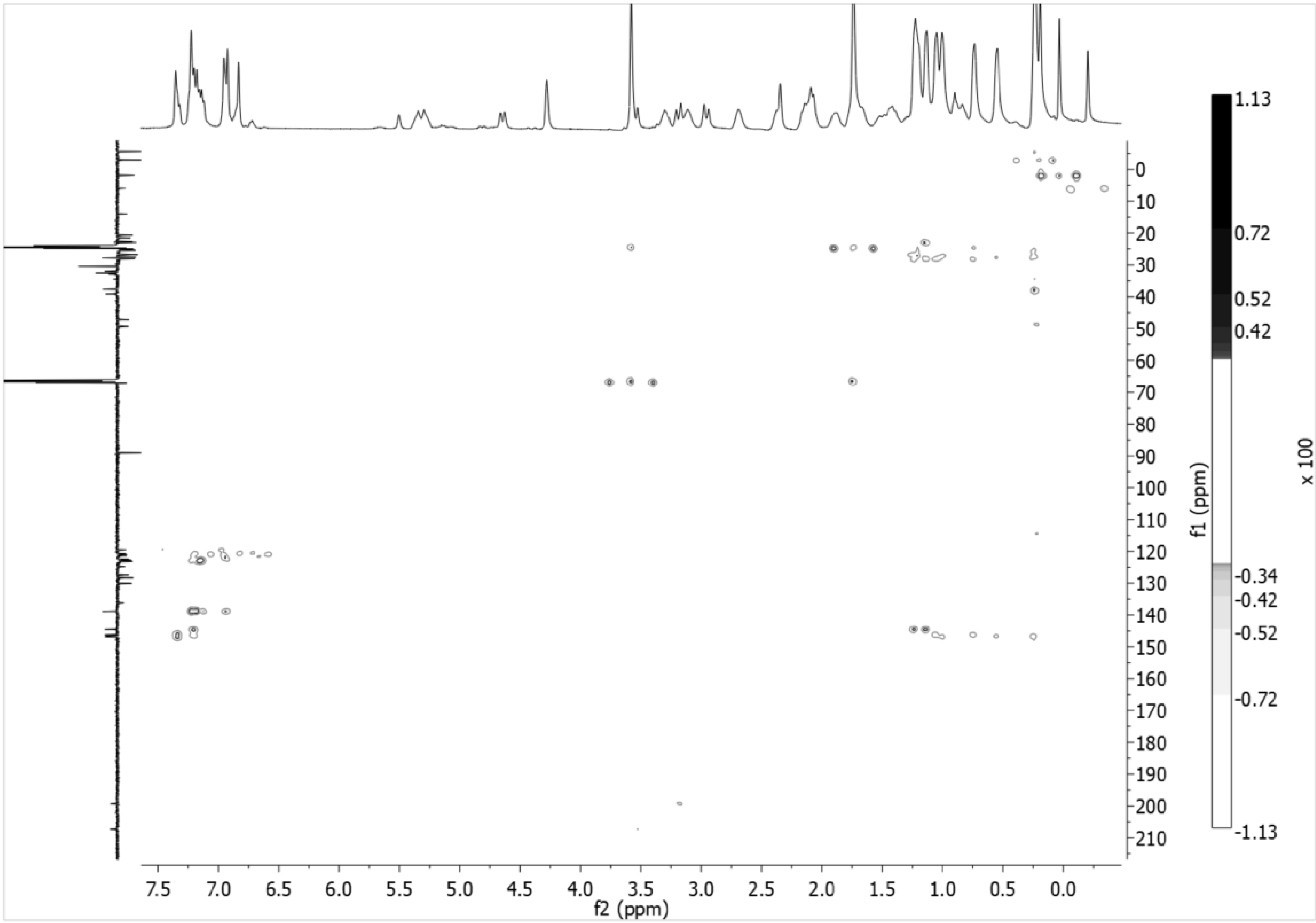

Figure S18. ^{1}H-^{13}C HMBC spectrum of **2** (400 MHz, 195 K, THF-d_8).

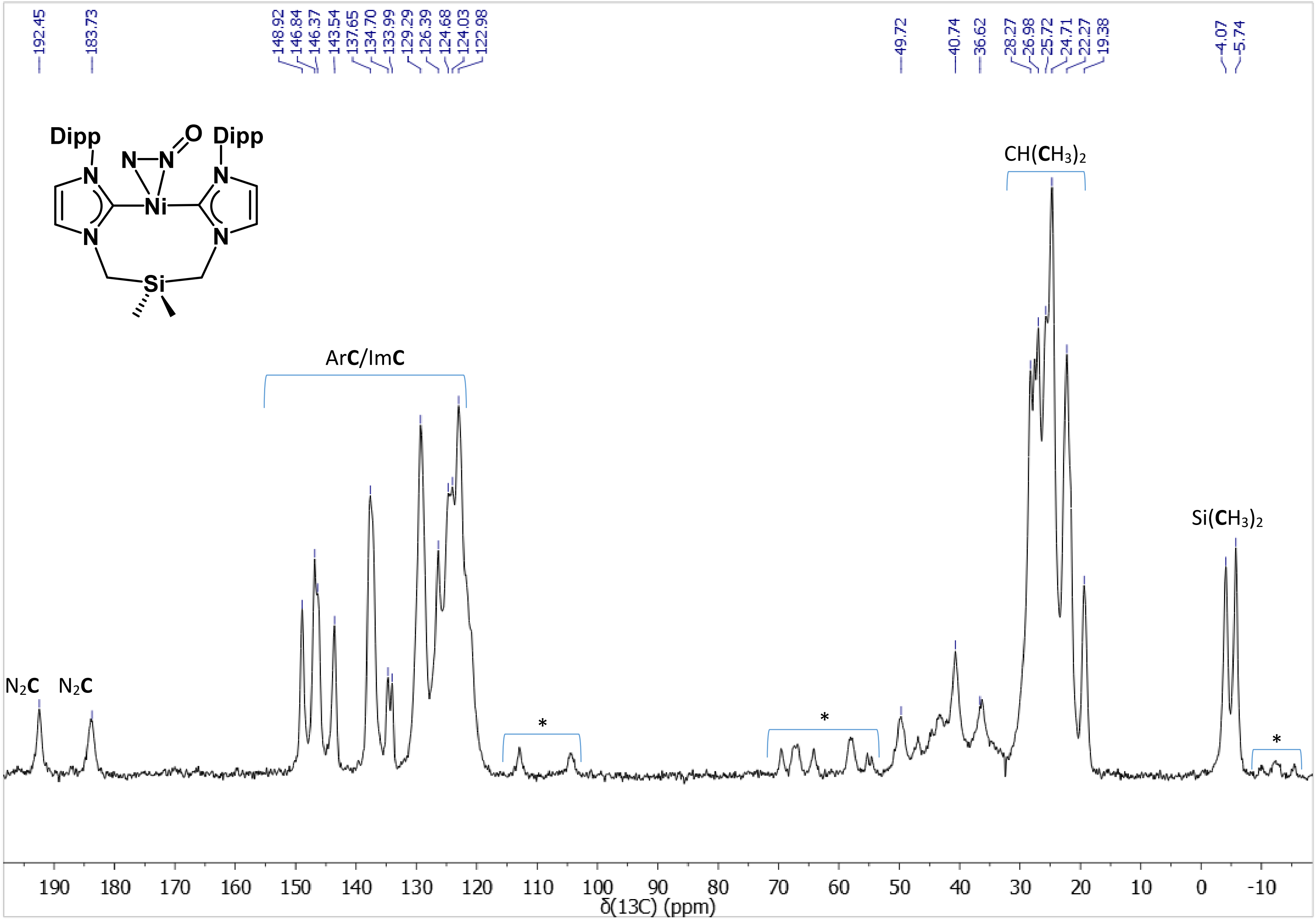

Figure S19. ^{13}C CP/MAS NMR spectrum of **3**, ν_{rot} = 10 kHz, acquired at 225 K at 11.75 T. (*) indicates spinning sidebands.

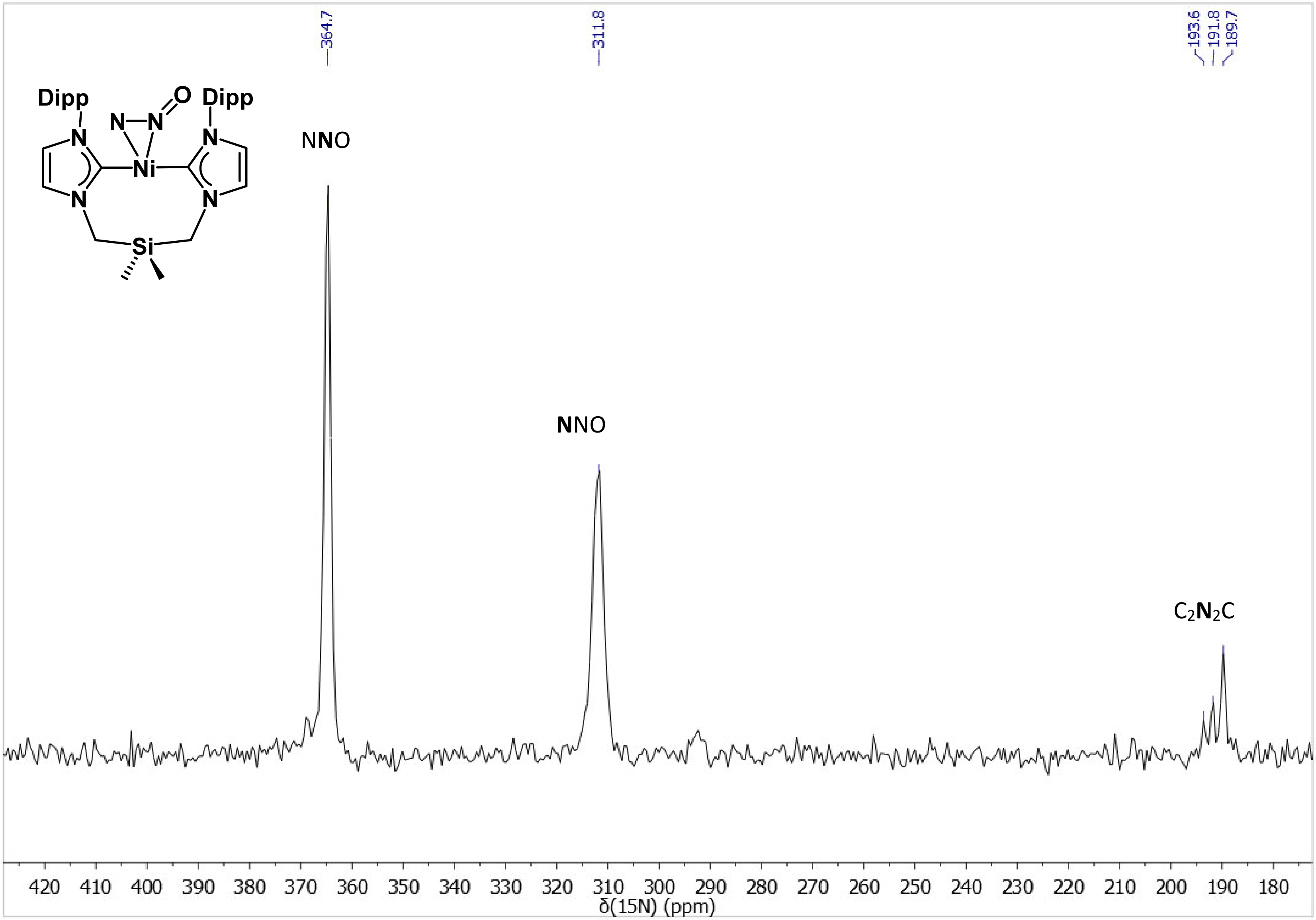

Figure S20. ^{15}N CP/MAS NMR spectrum of **3**, acquired at 225 K at 11.75 T with ν_{rot} = 10 kHz.

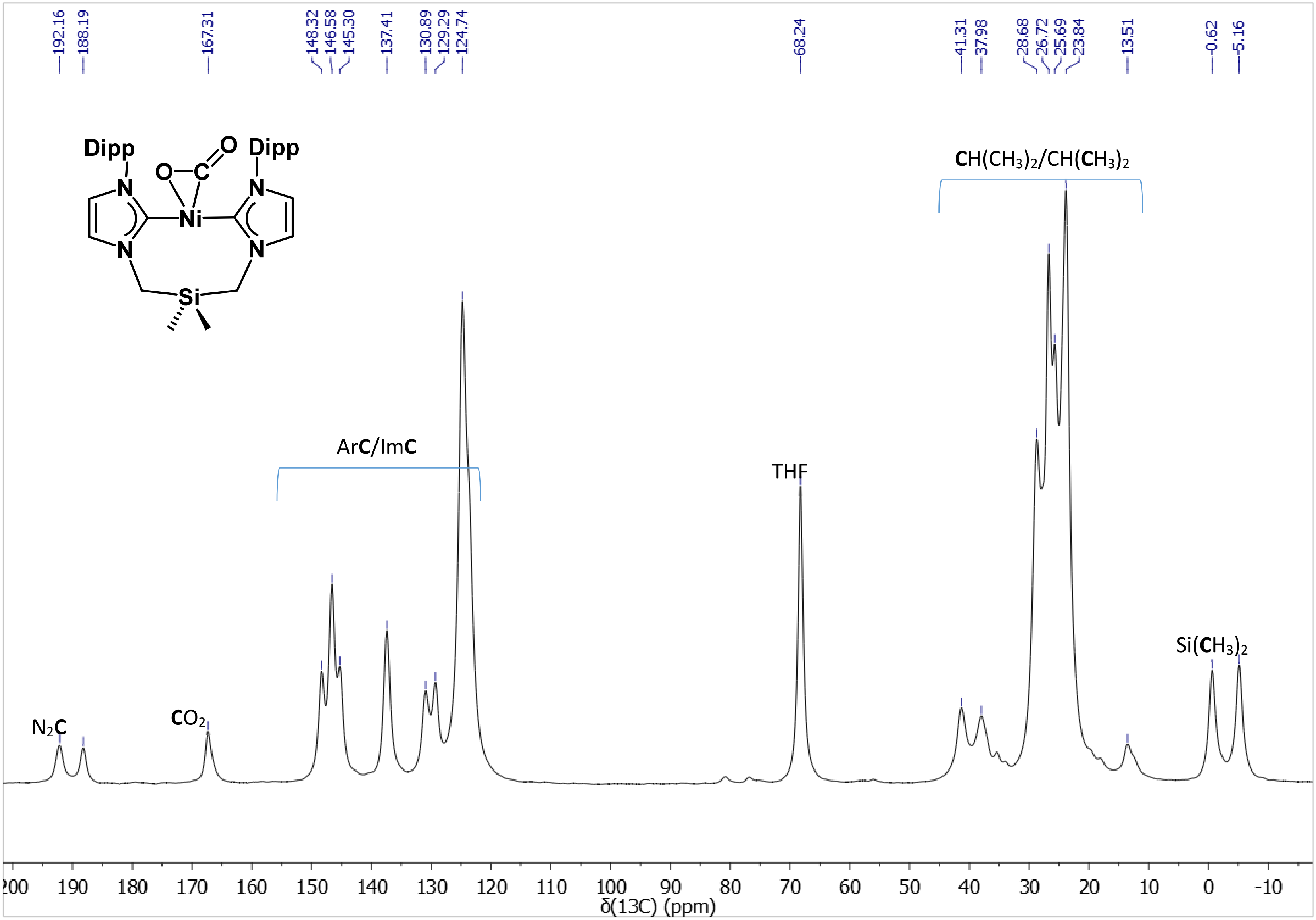

Figure S21. ^{13}C CP/MAS NMR spectrum of **5**, acquired at RT at 11.75 T with ν_{rot} = 14 kHz.

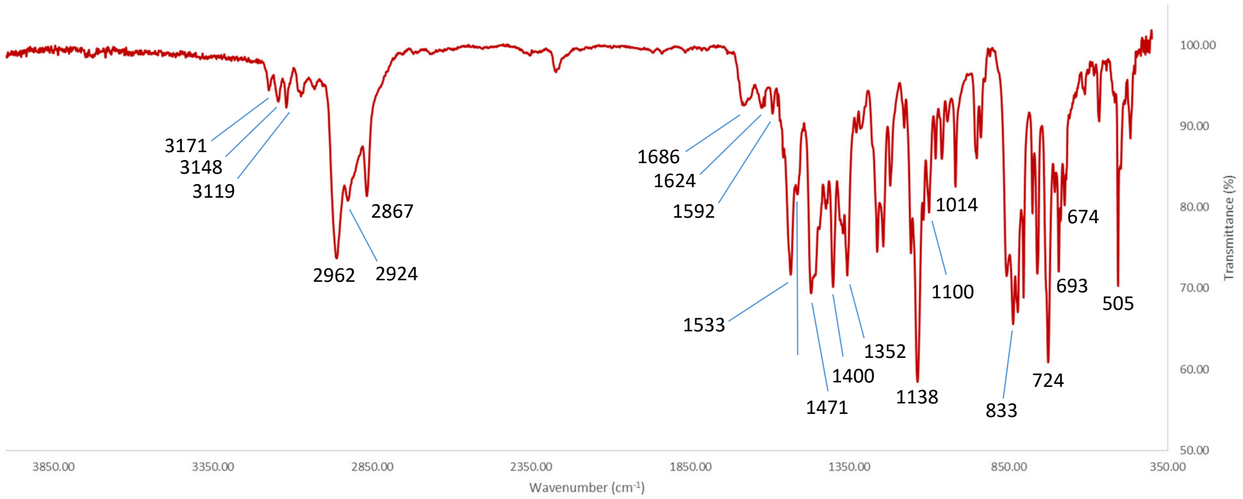

Figure S22. Baseline-corrected FT-IR for $^{14}N_2$-**3** at RT with KBr pellet.

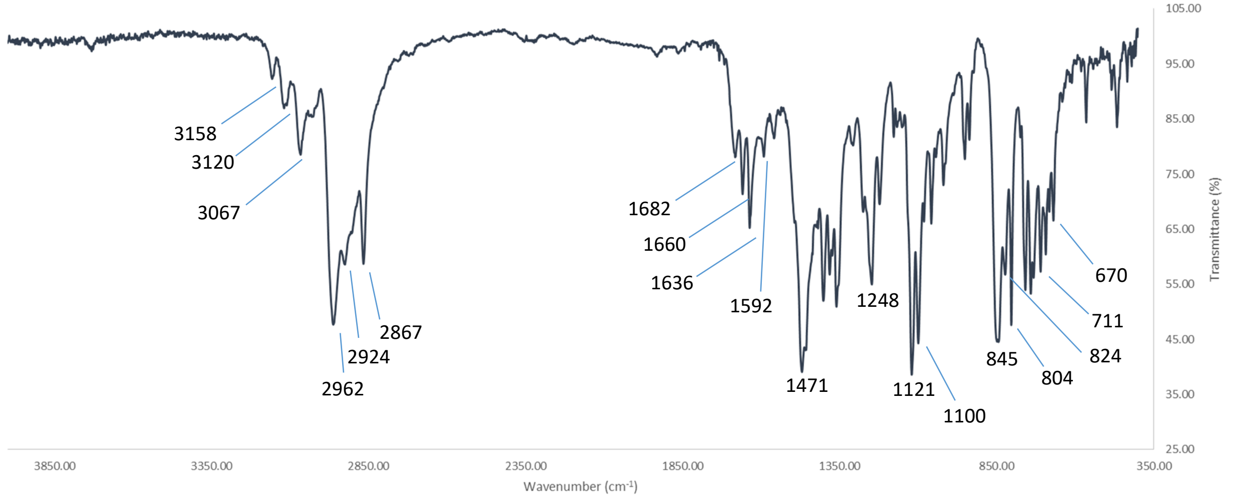

Figure S23. Baseline-corrected FT-IR for isotopically-labelled $^{15}N_2$-**3** at RT with KBr pellet.

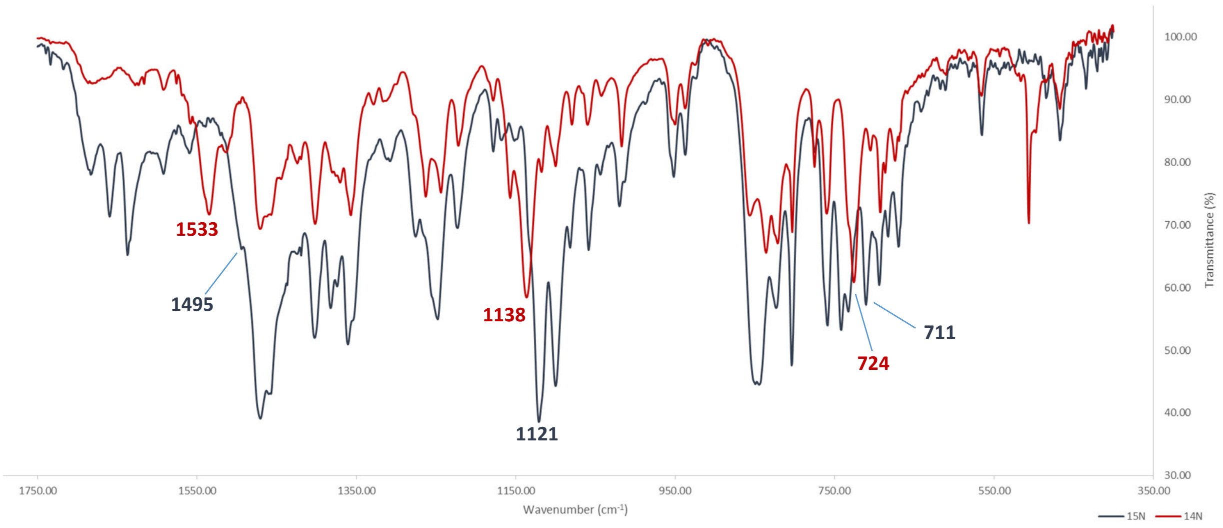

Figure S24. Superimposed inset of $^{15}N_2$-**3** (gray) and $^{14}N_2$-**3** (red).

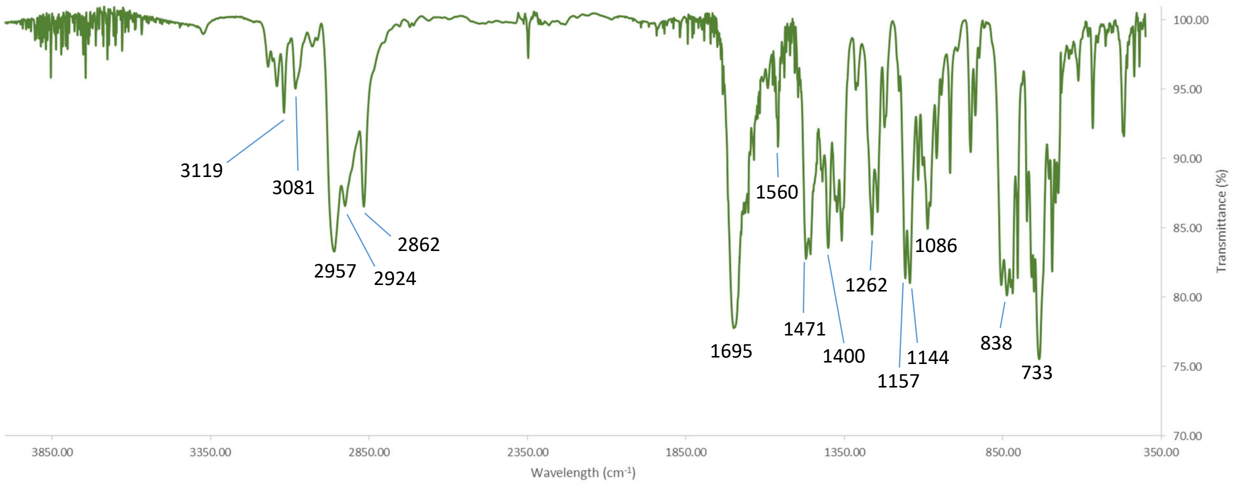

Figure S25. Baseline-corrected FT-IR for **5** at RT with KBr pellet.

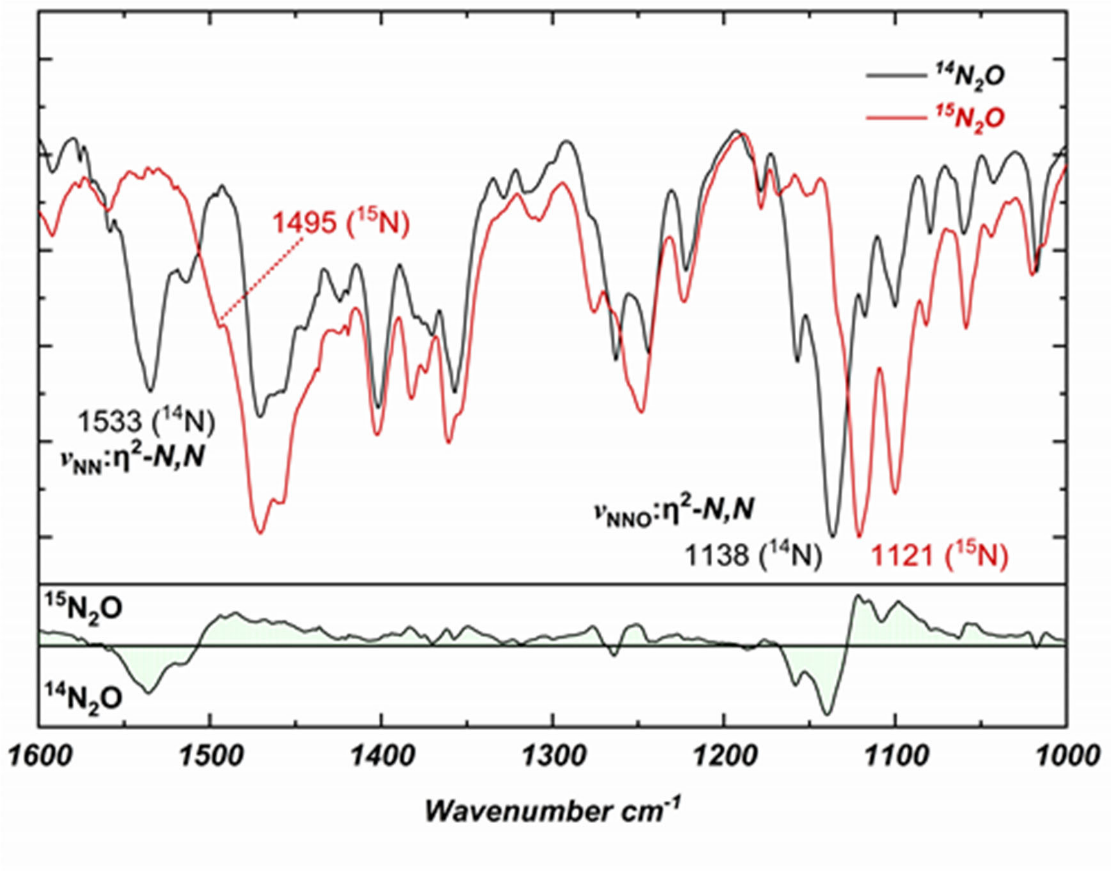

Figure S26. Overlaid FT-IR spectra for **3** and **3**-($^{15}N_2$) (99% isotopically enriched) with spectral difference below.

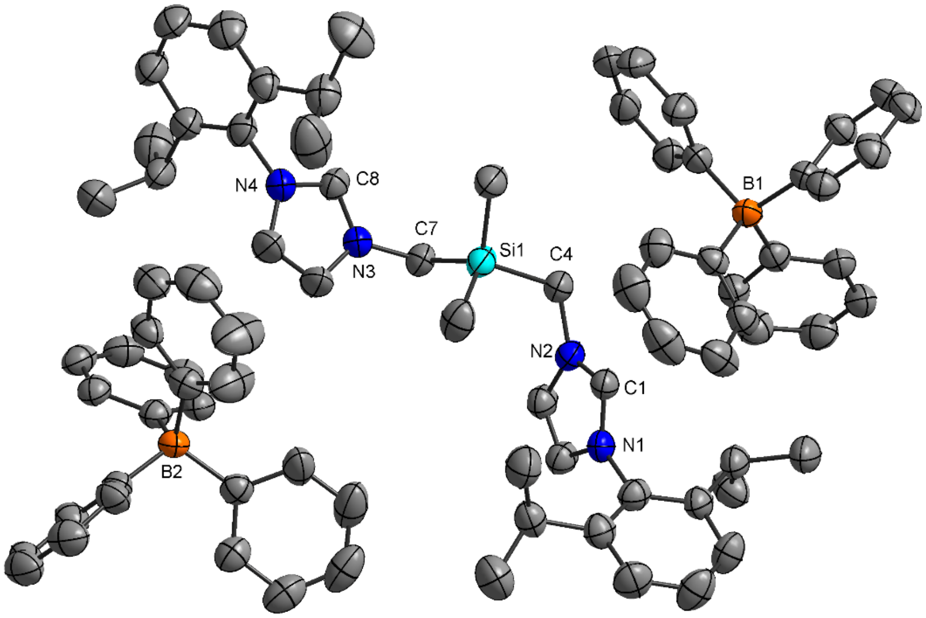

Figure S27. Solid-state structure of [(**1**)H_2][BPh_4]$_2$ with thermal ellipsoids at 50% probability. All hydrogen atoms have been omitted for clarity. Selected bond distances (Å) and angles (°): C4-Si1 1.891(3), C7-Si1 1.895(4), C4-N2 1.483(4), C7-N3 1.481(4), N2-C4-Si1 113.1(2), N3-C7-Si1 111.3(2).

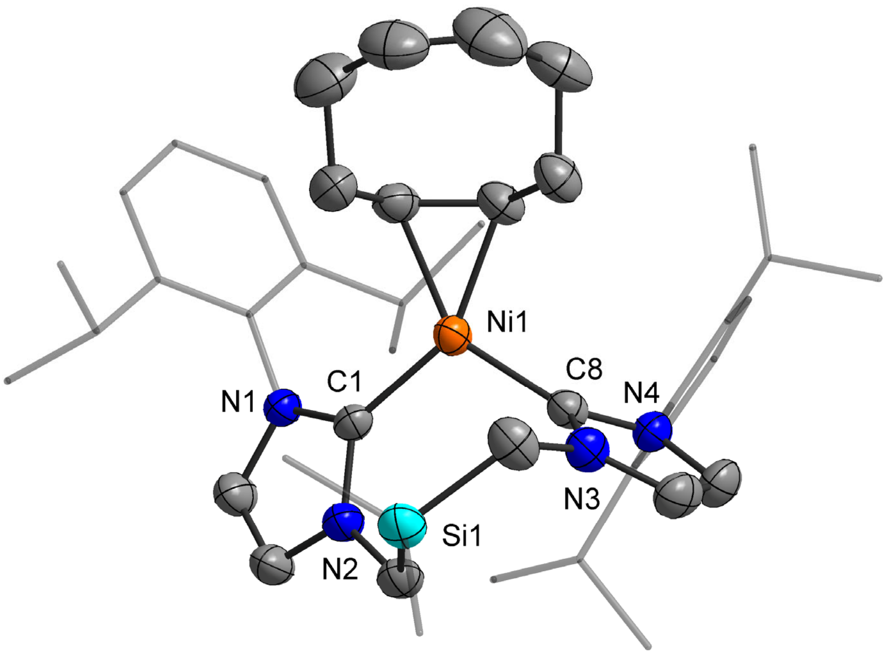

Figure S28. Solid-state structure of **2** with thermal ellipsoids drawn at 50% probability and all hydrogen atoms omitted for clarity. Selected bond distances (Å) and angles (°): Ni1-C1 1.900(3), Ni1-C8 1.879(3), C1-Ni1-C8 107.99(12), C4-Si1-C7 111.31(15).

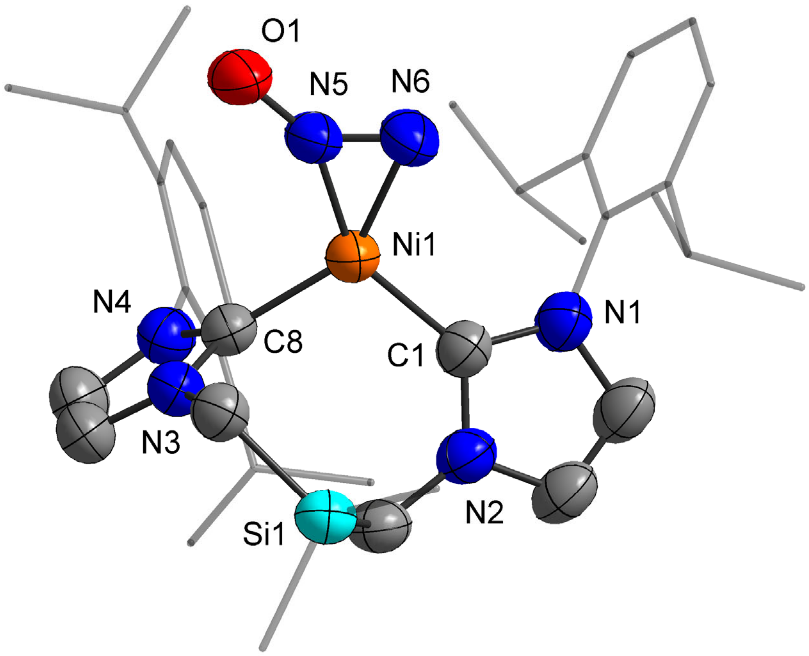

Figure S29. Solid-state structure of one of the two independent molecules of **3** with 50% thermal ellipsoids, and hydrogen atoms omitted. Selected bond lengths (Å) and angles (°) with [calculated values]: N5-N6 1.225(4) [1.210], N5-O1 1.276(4) [1.239], Ni1-N5 1.803(3) [1.821], Ni-N6 1.926(3) [1.910], Ni1-C1 1.901(3) [1.934], Ni1-C8 1.893(3) [1.919], N5-N6-O1 134.7(3) [138.4], C1-Ni1-C8 104.47(13) [107.7].

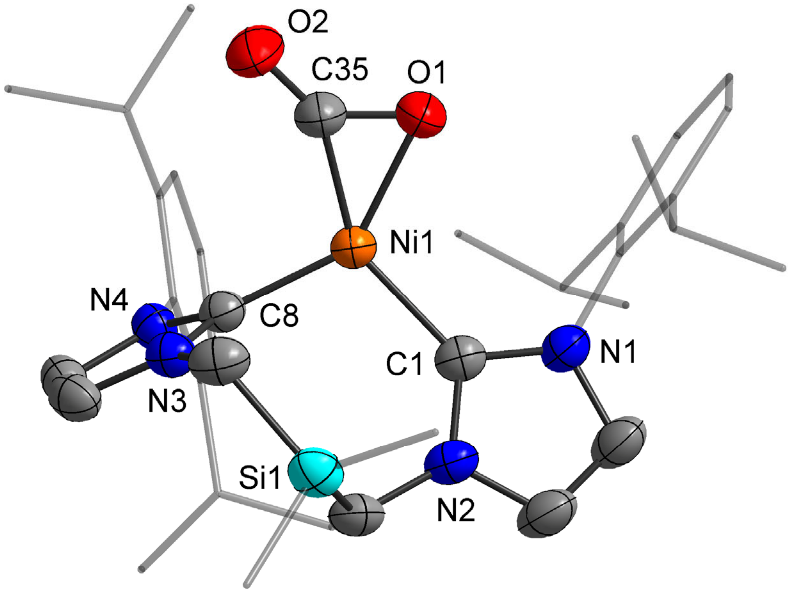

Figure S30. Solid-state structure of **5** with 50% thermal ellipsoids, and hydrogen atoms omitted. Selected bond lengths (Å) and angles (°) with [calculated values]: O1-C35 1.283(4) [1.264], C35-O2 1.218(4) [1.206], Ni1-O1 1.949(2) [1.937], Ni-C35 1.828(3) [1.839], Ni1-C1 1.973(3) [1.964], Ni1-C8 1.866(2) [1.856], O1-C35-O2 134.6(3) [137.5], C1-Ni1-C8 106.95(10) [109.4].

Table S1. Summary of Crystallographic Data for Compounds [(**1**)H_2][BPh_4], **2**, **3**, and **5**.

	[(**1**)H_2][BPh_4]$_2$	**2**	**3**	**5**
CCDC Deposition #	2023414	2023411	2023412	2023413
Formula	$C_{82}H_{90}B_2N_4Si$ + [solvent]	$C_{84}H_{120}N_8Ni_2Si_2$	$C_{100}H_{136}N_{12}Ni_2O_2Si_2$	$C_{39}H_{56}N_4NiO_3Si$
Formula weight	1181.28	1415.47	1711.80	715.67
Crystal system	Orthorhombic	Monoclinic	Triclinic	Orthorhombic
Space group	P b c a	P 2_1/c	P -1	P b c a
a (Å)	13.9281(6)	22.7004(6)	14.5896(4)	19.9519(6)
b (Å)	17.0348(7)	21.3563(6)	16.7064(5)	16.5247(4)
c (Å)	62.930(2)	17.5956(4)	20.5643(5)	23.7078(6)
α (°)	90	90	90.901(2)	90
β (°)	90	109.6770(10)	95.122(5)	90
γ (°)	90	90	97.415(2)	90
Volume ($Å^3$)	14930.8(10)	8032.2(4)	4948.7(2)	7816.4(4)
Z	8	4	2	8
Temperature (K)	173	173	173	173
λ (Å)	1.54178	1.54178	1.54178	1.54178
ρ(calc) (gcm^{-1})	1.051	1.171	1.149	1.216
F(000)	5072.0	3056.0	1840.0	3072.0
R(int)	0.0809	0.0411	0.0509	0.0390
μ (mm^{-1})	0.599	1.219	1.100	1.318
θ range (°)	2.808–139.908	4.134–140.18	4.316–140.374	7.458–136.724
Total data	48979	51661	18654	47395
Unique	13924	15168	18654	7160
Completeness (%)	98.5	99.5	99.0	99.8
Parameters	812	885	1169	443
R (>2σ)	0.0836	0.0670	0.0641	0.0501
R_w (all data)	0.2385	0.1945	0.2068	0.1485
GOF	1.002	1.033	1.031	1.064

Deposition Numbers 2023411-2023414 contain the supplementary crystallographic data for this paper. These data are provided free of charge by the joint Cambridge Crystallographic Data Centre and Fachinformationszentrum Karlsruhe Access Structures service www.ccdc.cam.ac.uk/structures.

Computational Details

All calculations were performed with Gaussian 16-C.01[16] (geometry optimizations, energetics and NMR) and ADF 2019.3[17,18] (NMR and energy decomposition analyses) program suites.

Geometries and properties of complexes 3, 4 and 5. Geometries of **3**, **4** and **5** were optimized in the gas phase with density functional theory using the PBE1PBE functional,[19,,21] def2-TZVP[22] basis sets and Grimme's D3 dispersion correction with Becke-Johnson damping.[23,24] All structures were confirmed to be minima on the potential energy hypersurface via calculation of the associated vibrational frequencies (all positive).

The potential energy surfaces of **3** and **4** were examined in more detail by locating the transition states (one imaginary frequency) for their formation from (**1**)Ni and N_2O (relative Gibbs energy set to 0 kJ mol^{-1}, Figure S31). The results show that the associated energy barriers are both relatively low, with the transition state (**1**)Ni···NNO being lower by 32 kJ mol^{-1}, consistent with **3** being a kinetic product. The η^2-*N*,*N*'-bound species **3** is 54 kJ mol^{-1} lower in energy than the η^2-*N*,*O*-isomer **4**, as expected, but **4** is metastable and readily converts to (**1**)Ni(N_2)O almost without a barrier (all attempts to locate the associated transition state were unsuccessful and converged to (**1**)Ni(N_2)O instead). Thus, (**1**)Ni(N_2)O is the thermodynamic product, although only by 7 kJ mol^{-1}. Pathways connecting **3** and **4** or **3** and (**1**)Ni(N_2)O were not searched for on the potential energy surface.

Calculated ^{15}N chemical shifts of 3. ^{15}N NMR shielding tensors were calculated for **3** using the GIAO formalism[25] at the PBE1PBE/def2-TZVP level[19–22] with the Gaussian program.[16] For comparison and inclusion of scalar-relativistic effects, the shielding tensors were also calculated using the ADF program[18] employing the BP86[26,27] density functional in combination with frozen-core TZ2P[28] basis sets and zeroth order regular approximation (ZORA).[29] Shielding values were then converted to chemical shifts based on the reported absolute nitrogen shielding for NH_3(l) of 244.6 ppm (*vide infra*), *i.e.*, $\delta_{calc} = 244.6 - \sigma_{calc}$.[30] For better match with the experimental, solid state, conditions, atomic coordinates determined for **3** via X-ray crystallography were used in NMR calculations, apart from the C–H bond lengths, which were set to 1.09 Å.

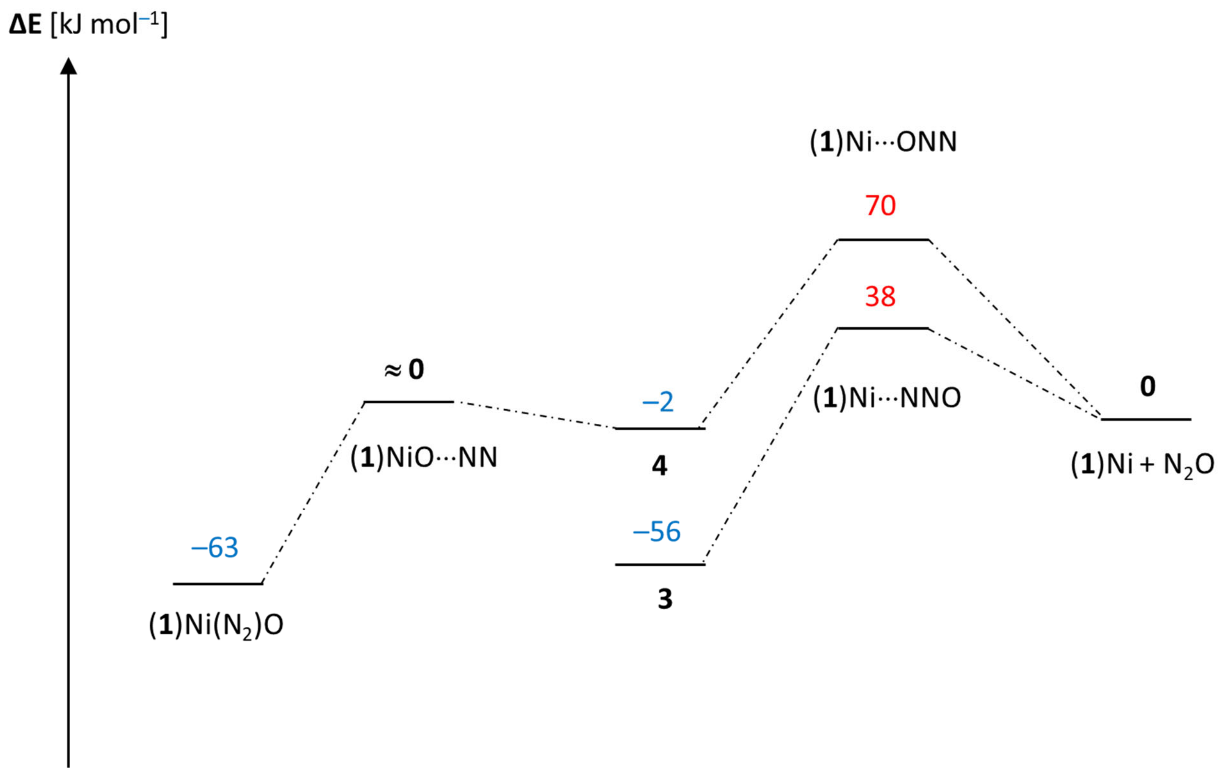

Figure S31. Relative Gibbs energies for **3**, **4** and (**1**)Ni(N_2)O, and the associated transition states connecting the three species.

Comparison of ligand properties of N_2O and CO_2. To compare the binding of ligands N_2O and CO_2 to metal complexes (M), Gibbs energies were calculated for the ligand exchange reaction M-CO_2 + N_2O ⇌ M-N_2O + CO_2 using 12 crystallographically characterized η^2-*C*,*O*-CO_2 complexes (Figure S32) and their hypothetical η^2-*N*,*N'*-N_2O analogues. The geometries of the complexes were optimized with the same method as used for **3** and **5** to allow direct comparison of the results. The calculated energies (Table S2) show that in most cases, N_2O binds stronger to the metal, with differences up to –26 kJ mol^{-1} (per molecule of ligand). These results are consistent with the morphology and energies of the frontier molecular orbitals of N_2O and CO_2 (Figure S33), that is, the LUMO of N_2O is lower in energy than that of CO_2, and has greater contribution from the terminal nitrogen rather than the oxygen atom. It needs to be stressed that these results do not imply that the studied η^2-*N*,*N'*-N_2O complexes are stable or isolable species but merely show that the inherent π-acceptor nature of N_2O is comparable to, or even better than, that of CO_2.

To further analyze the metal-ligand interactions in complexes of CO_2 and N_2O, the bonding in **3** and **5** was investigated through energy decomposition analysis with the extended transition state method using natural orbitals for chemical valence (ETS-NOCV).[31] These were performed with the ADF program[18] at the PBE1PBE/TZ2P/ZORA[19–21,28,29] level of theory. The results showed that in both cases, the Pauli repulsion (769 and 729 kJ mol^{-1} for **3** and **5**, respectively) is significantly greater than the sum of electrostatic attraction (–454 and –464 kJ mol^{-1}) and dispersion (–22 and –24 kJ mol^{-1}). However, the calculated instantaneous interaction energies are vastly negative for both species, –415 and –402 kJ mol^{-1} for **3** and **5**, respectively, owing to efficient orbital interactions (–709 and –643 kJ mol^{-1}) of which the vast majority (approx. 80 % in both cases) comes from a single orbital pair, consistent with a charge transfer channel from the metal to the π* system of the ligand. It should be stressed that a comparison between **3** and **5** shows comparable electrostatic attraction and dispersion terms, but significantly stronger orbital interactions in **3** (difference of 66 kJ mol^{-1} compared to **5**), in line with the better π-acceptor nature of N_2O in these systems. As a result, the calculated instantaneous interaction energy is 13 kJ mol^{-1} more negative for **3** than **5**, in harmony with the trend in calculated binding energies that also take into account relaxation of the geometries of the ligands and (**1**)Ni.

Table S2. Gibbs Energies Calculated for the Complexes M-CO_2 (Figure S32) and M-N_2O (in a.u.), and for Ligand Exchange Reaction M-CO_2 + N_2O ⇌ M-N_2O + CO_2 (in kJ mol^{-1}) Using Different Metal Fragments M.[a]

**Metal**	**CCDC code**	**E(M-CO_2 + N_2O)**	**E(M-N_2O + CO_2)**	**ΔG**
Ni	OXEXUL[32]	–3272.616904	–3272.626824	–26
	LEKXIK[33]	–3273.804873	–3273.813608	–23
	LONFUR[34]	–3879.81288	–3879.808607	11
	**5**, this work	–3712.226853	–3712.235493	–23
Mo	CUSYAQ[35]	–827.900267	–827.906703	–17
	IFAPIR[36]	–2289.621949	–2289.621018	2
	SIBQAX[37]	–1538.183193	–1538.191927	–23
	FELGEJ[b, 38]	–2329.377073	–2329.403074	–34
Nb	BAHXUD[39]	–1343.446634	–1343.449893	–9
	JUFWEM[40]	–1165.860093	–1165.86454	–12
Fe	HEHKIO[41]	–3791.418731	–3791.422571	–10
Re	NAZKUU[42]	–970.261935	–970.259059	8
	SABXEZ[43]	–1163.196807	–1163.193362	9

[a] Energies of CO_2 and N_2O are –188.476081 and –184.556057 a.u., respectively.
[b] Complex includes two ligands *i.e.* formulae M-2CO_2 and M-2N_2O.

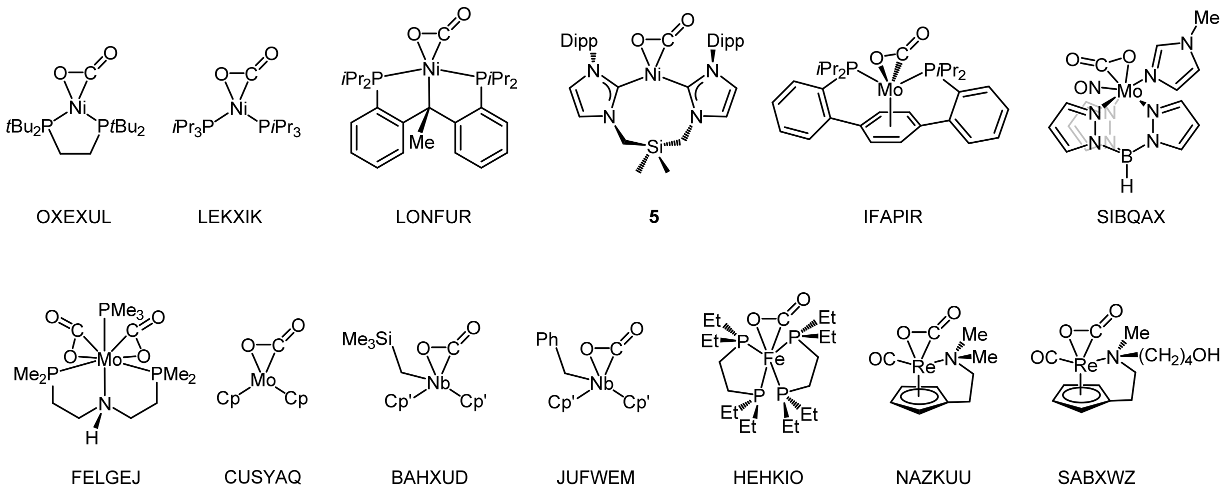

Figure S32. M-CO_2 complexes used for the computational study summarised in Table S2.

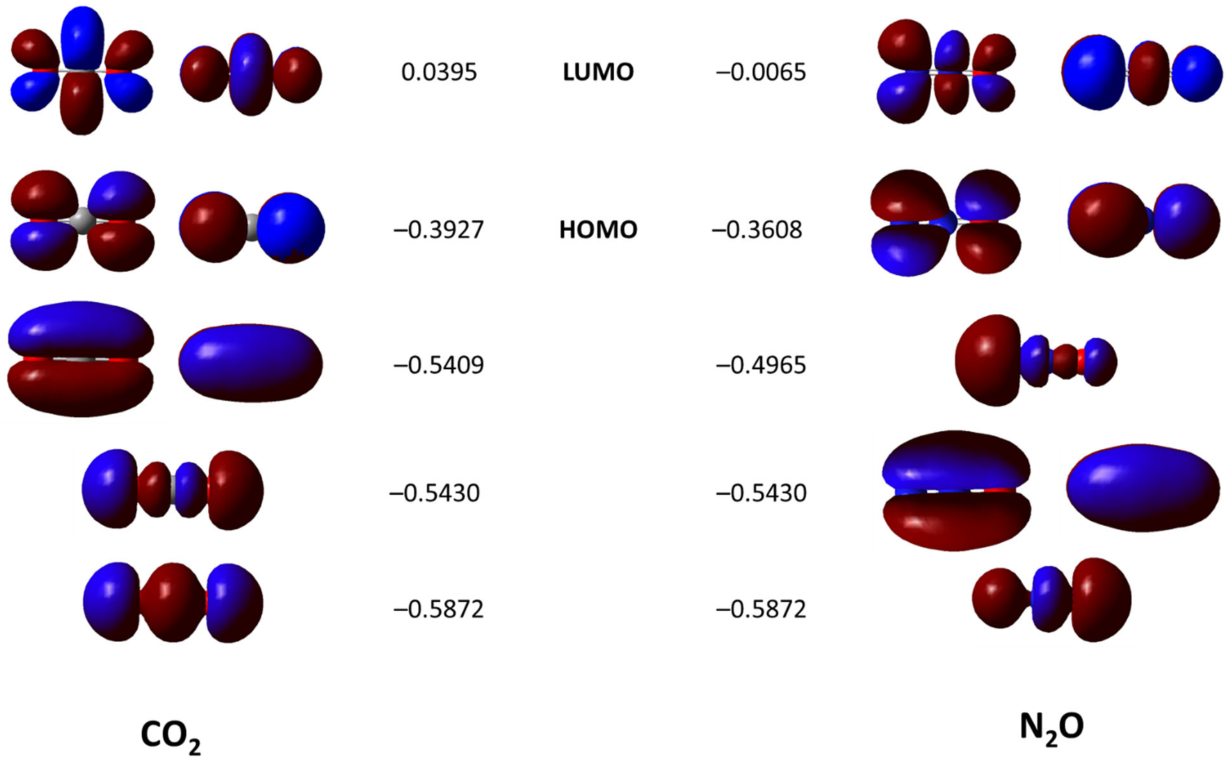

Figure S33. Frontier orbitals of CO_2 and N_2O (PBE1PBE/def2-TZVP, isosurface value ±0.03), and their associated energies (in a.u.).

Table S3. Summary of Results (in kJ mol^{-1}) from Energy Decomposition Analyses Conducted for **3** and **5**.

	**3**	**5**
$\Delta E_{\mathrm{Pauli}}$	769	729
$\Delta E_{\mathrm{elstat}}$	–454	–464
ΔE_{orb}	–709	–643
ΔE_{disp}	–22	–24
ΔE_{int}	–415	–402

Solid-State NMR

^{13}C and ^{15}N NMR spectra of solid samples were acquired at room temperature (**5**) or at a temperature of approximately 225 K (**3**) on a Bruker NEO 500 spectrometer, operating at 125.8 and 50.7 MHz, respectively, for ^{13}C and ^{15}N. The cross-polarization technique, with magic angle spinning at frequencies (ν_{rot}) of 9.5 to 14 kHz, was used to acquire all spectra of solid samples. The ^{1}H 90° pulse was 4.0 μs; the contact times were 3.0 and 5.0 ms respectively for ^{13}C and ^{15}N, with acquisition times of 50 and 20.5 ms, respectively, and with recycle delays of 3.0 or 5.0 s, respectively, for these nuclei. ^{13}C NMR spectra were referenced to TMS (δ = 0) by setting the high-frequency peak of an MAS adamantane sample to 38.56 ppm;[44] those for ^{15}N were referenced to NH_3 (δ = 0 ppm) by setting the ^{15}N peak for glycine-^{15}N (99 %) to 33.4 ppm.[45]

Referencing nitrogen chemical shifts. Many early nitrogen NMR studies used ^{14}N as the NMR probe instead of ^{15}N. ^{14}N has a natural abundance 99.632 %, a relatively low frequency ratio, Ξ (^{14}N) = 7.226317 %, and a spin quantum number of I = 1 with a relatively small quadrupole moment, Q = 2.044 fm^2. Typically, ^{14}N nuclear quadrupolar coupling constant values, C_q, are less than 5 MHz and consequently for relatively small molecules (*e.g.*, N_2O, acetonitrile) ^{14}N NMR linewidths in non-viscous solvents may be only a few Hz or less. Much of the early ^{14}N NMR research in organic chemistry was done at the Institute of Organic Chemistry, Polish Academy of Sciences in Warszawa, Poland. The relative receptivity of ^{14}N compared to ^{13}C is 5.90; however, one has to keep in mind that for large molecules in solution significant line broadening is encountered because of efficient quadrupolar relaxation. In 1977, Witanowski et al. proposed neat nitromethane, CH_3NO_2, as a reference sample for nitrogen NMR (*i.e.*, δ = 0.00 ppm at 30 °C).[46] In that paper nitrogen chemical shifts are given as "screening constants"; a positive screening constant means that the chemical shift is negative. For example, for neat liquid ammonia at 30 °C, NH_3, the screening constant is +381.93 ± 0.14 ppm (*i.e.*, δ = –381.93 ± 0.14 ppm). Early nitrogen NMR studies were discussed in Witanowski's 1973 book "Nitrogen NMR",[47] as well as in several reviews by the same author.[48] In Table 2, pp. 86–87 of the most recent report, the "true" nitrogen shielding of neat liquid ammonia is given as 381.9 ppm relative to CH_3NO_2.[48e] For the applied magnetic field, $\mathbf{B}_0$, perpendicular to the sample, the number reported is 380.2 ppm.

With the availability of pulse Fourier transform NMR instrumentation in the 1970s, natural abundance ^{15}N NMR studies became feasible. Nitrogen-15 has a larger magnetic moment than ^{14}N,

Ξ (^{15}N) = 10.136767 %, (*i.e.*, $\gamma(^{15}N)/\gamma(^{14}N) = -1.40276$). Of note, ^{15}N has a negative magnetogyric ratio, which is important to recognize in phasing ^{15}N NMR spectra of liquid samples. In 1977, Srinivasan and Lichter reported $^{15}NH_3$(*liq*.), $\delta_{iso} = -380.23$ ppm relative to CH_3NO_2 (measurements at room temperature).[49] A turning point in nitrogen NMR spectroscopy was the publication of Levy and Lichter's book "Nitrogen-15 Nuclear Magnetic Resonance Spectroscopy" in 1979.[50] It used liquid ammonia as a reference, which may be less confusing because nitrogen sites in most samples are less shielded than the nitrogen nuclei of ammonia (*i.e.*, most sites have positive chemical shifts). However, the IUPAC 2001 recommendations continue to use CH_3NO_2 as a reference for nitrogen chemical shifts.[51]

Of course, nitrogen chemical shifts can be expressed using either CH_3NO_2(l) or NH_3(l) as a reference. Duncan's "Principal Components of Chemical Shift Tensors: A Compilation", is a reliable source for solid-state NMR data on chemical shift tensors,[52] which follows the IUPAC convention setting the nitrogen chemical shift of NH_3 at –380 ppm. For an overall introduction to nitrogen NMR, we recommend the *Encyclopedia of NMR*.[53] Also recommended are Mason et al.'s review on metal nitrosyls and related compounds[54] and von Philipsborn et al.'s early review on ^{15}N NMR.[55]

Referencing nitrogen chemical shifts in solids. The first systematic report of ^{15}N chemical shifts for nitrogen in various ammonium salts appeared in 1991 and included NH_4Cl(s), $\delta = -341.17$ ppm; NH_4NO_3, $\delta = -358.45$ ppm for NH_4^+ and –4.98 ppm for NO_3^-; glycine, –347.58 ppm, relative to neat CH_3NO_2(l).[56] The most recent paper focusing on ^{15}N referencing in solids reflected the fact that in biochemical applications liquid ammonia is the more common reference (see refs. 16–18 of this paper) and recommend using the unified scale which is Ξ (^{15}N) = 10.132912 % instead of Ξ (^{15}N) = 10.136767 % (IUPAC).[45] According to these values, in an applied magnetic field where the ^{1}H resonance of TMS is exactly 100.0 MHz, the ^{15}N of liquid ammonia is 10.132 912 MHz and that of CH_3NO_2 is 10.136 767. Thus, the ^{15}N chemical shift of CH_3NO_2 is reported by Bertani as 136,767 Hz – 132,912 Hz = 3855/10.132 912 = 380.44 ppm. The values used in the current manuscript stem from this paper.

Depending on the exact experimental conditions (temperature, with or without MAS, etc.) the chemical shift values for glycine vary slightly.[57] A chemical shift reported at –6.4 ppm from NH_4Cl, which has a chemical shift of 39.3 ppm with respect to NH_3, yields a value for glycine of

approximately 39.3 – 6.4 = 32.9 ppm. α-glycine was reported as a secondary reference at −346.6 ppm,[58] similar to the value reported by Hayashi and Hayamizu (−347.58 ppm).[56]

NMR of nitrous oxide. In a paper that established an absolute shielding scale for nitrogen it was reported σ(N) = 244.6 ppm for NH_3 (liq., 298 to 303 K) and −135.8 ppm for CH_3NO_2 (liq., 298 to 303 K), resulting in a shielding difference of 380.4 ppm.[30] Values for a few other samples are given including N_t=N_c=O neat liquid at 193 K: σ(N_t) = 90.2 ppm and σ(N_c) = +6.2 ppm. The corresponding shielding values in the gas phase at 300 K were estimated as 99.5 and 11.3 ppm. Taking the nitrogen chemical shift of liquid ammonia as 0.0 ppm, this data yields N_2O (liq. at 193 K), δ_t = 154.4 ppm (*i.e.*, σ(N_t) = 90.2 ppm) and δ_c = 238.4 ppm (*i.e.*, σ(N_c) = 6.2 ppm). For N_2O in the gas phase at 300 K, it was concluded that σ(N_t) = 99.5 ppm and σ(N_c) = 11.3 ppm; hence δ(N_t) = 145.1 ppm) and δ(N_c) = 233.3 ppm. In CCl_4 solution at 303 K, σ(N_t) = 96.5 ppm (δ(N_t) = 148.1 ppm) and σ(N_c) = 12.2 ppm (δ(N_c) = 232.4 ppm),[59] while in CD_3CN, δ(N_t) ≈ 149 ppm (*i.e.*, 380 – 231) and δ(N_c) ≈ 233 ppm (*i.e.*, 380 – 147), where isotope shifts and spin-spin coupling constants have also been determined.[60] The nitrogen shielding of NH_3 is sensitive to whether it is in the gas or liquid phase; in the gas phase the nitrogen nucleus is more shielded by approximately 20 ppm (the same is true for the oxygen of water). In the gas phase, the nitrogen nuclei of N_2O are also more shielded than in the liquid phase but the difference is less than 10 ppm for the terminal nitrogen and 6 ppm for the central nitrogen.

Table S4. Experimental and Calculated Isotropic Chemical Shifts (ppm) for **3**.[a,b]

Nucleus[b]	Exptl.	PBE1PBE	BP86
$N_{central}$	364.7	395	366
N_{term}	311.8	313	296
N1, N2, N3, N4	190 – 194	183 – 203	186 – 203

[a] Calculated magnetic shieldings were converted to chemical shifts according to $\delta_{calc} = 244.6 - \sigma_{calc}$.[30]

[b] See manuscript for atom labelling.

For linear molecules such as N_2O the paramagnetic shielding along the bond axis is zero. When the N_2O ligand is bent, the paramagnetic term, which is negative, becomes important and all three nuclei of nitrous oxide are deshielded relative to when it is linear.

Table S5. PBE1PBE (top) and BP86 (bottom) Calculated Nitrogen and Oxygen Magnetic Shielding Tensors (ppm) for N_2O and **3**.[a,b]

	σ_{11}	σ_{22}	σ_{33}	σ_{iso}	Ω	κ
			**N_2O**			
O	76.7	76.7	416.8	190.1	340.1	1.0
	58.3	58.3	418.0	178.2	359.7	1.0
$N_{central}$	−169.4	−169.4	352.3	4.5	521.7	1.0
	−166.3	−166.3	353.9	7.1	520.2	1.0
N_{term}	−24.9	−24.9	343.8	98.0	368.7	1.0
	−29.4	−29.4	345.2	95.5	374.6	1.0
			**3**			
O	−350.0	−145.3	198.5	−99.0	548.5	0.253
	−282.6	−138.0	172.3	−82.8	454.9	0.364
N1	−71.8	61.0	133.8	41.0	205.6	−0.292
	−59.1	58.0	127.3	42.1	186.4	−0.256
N2	−64.9	95.5	151.3	60.7	216.2	−0.483
	−50.0	85.3	137.0	57.4	187.0	−0.447
N3	−73.7	99.9	157.8	61.3	231.5	−0.500
	−61.9	96.0	141.8	58.7	203.7	−0.550
N4	−81.7	66.6	141.2	42.0	222.9	−0.331
	−65.4	66.9	130.9	44.1	196.3	−0.348
$N_{central}$	−354.1	−148.9	52.1	−150.3	406.2	−0.012
	−287.5	−100.9	23.5	−121.6	311.0	−0.200
N_{term}	−268.7	−211.8	276.4	−68.0	545.1	0.791
	−232.7	−149.8	227.0	−51.8	459.7	0.639

[a] $\sigma_{iso} = \frac{1}{3}(\sigma_{11} + \sigma_{22} + \sigma_{33})$; $\Omega = \sigma_{33} - \sigma_{11}$; $\kappa = \frac{3(\sigma_{iso} - \sigma_{22})}{\Omega}$.

[b] See manuscript for atom labelling.

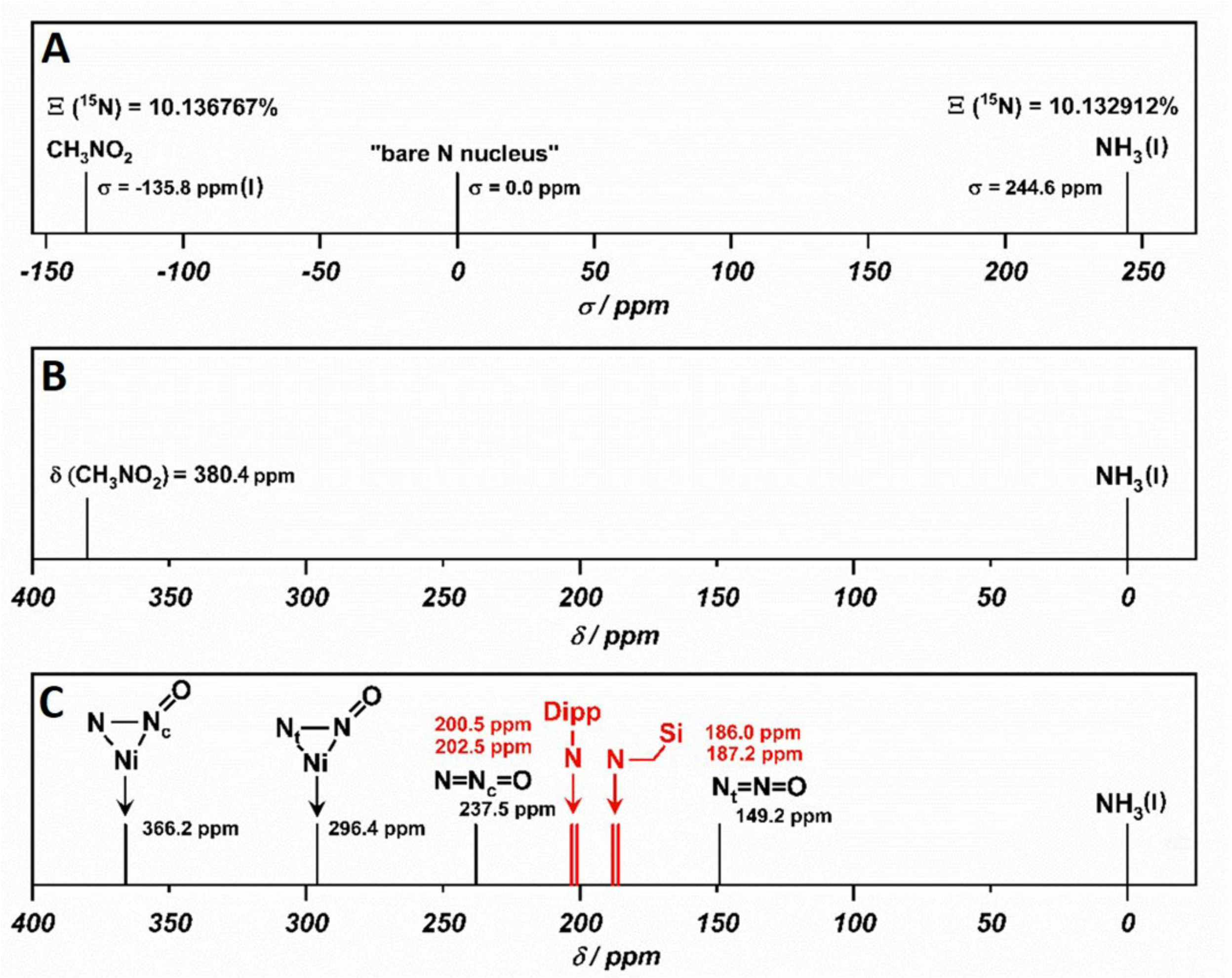

Figure S34 A) Absolute ^{15}N magnetic shielding values for NH_3 and CH_3NO_2. **B)** Conversion of shielding values to chemical shifts relative to NH_3(l) using the equation $\delta = + 244.6 - \sigma$ ppm. **C)**. Calculated (BP86/TZ2P/ZORA) ^{15}N chemical shifts for **3** and N_2O using σ(N) = 244.6 ppm for NH_3.

References

[1] G. R. Fulmer, A. J. Miller, N. H. Sherden, H. E. Gottlieb, A. Nudelman, B. M. Stoltz, J. E. Bercaw, K. I. Goldberg, *Organometallics* **2010**, *29*, 2176–2179.

[2] J. Liu, J. Chen, Y. Zhao, L. Li, H. A. Zhang, *Synthesis* **2003**, *17*, 2661–2666.

[3] S. Lee, Y. Jeon, Y. Lim, M. A. Hossain, S. Lee, Y. Cho, H. Ju, W. Kim, *Electrochim. Acta* **2013**, *107*, 675–680.

[4] J. R. Partington, *A History of Chemistry*, Vol. 3, MacMillan & Co., London, **1962**.

[5] M. R. Gyton, B. Leforestier, A. B. Chaplin, *Angew. Chem. Int. Ed.* **2019**, *58*, 15295–15298; *Angew. Chem.* **2019**, *131*, 15439-15442.

[6] D. J. Xiao, E. D. Bloch, J. A. Mason, W. L. Queen, M. R. Hudson, N. Planas, J. Borycz, A. L. Dzubak, P. Verma, K. Lee, F. Bonino, V. Crocellà, J. Yano, S. Bordiga, D. Truhlar, L. Gagliardi, C. M. Brown, J. R. Long, *Nat. Chem.* **2014**, *6*, 590–595.

[7] S. Chaturvedi, P. N. Dave, *J. Energ. Mater.* **2013**, *31*, 1–26.

[8] J. C. Oxley, J. L. Smith, S. Vadlamannati, A. C. Brown, G. Zhang, D. S. Swanson, J. Canino, *Propellants Explos. Pyrotech.* **2013**, *38*, 335–344.

[9] T. L. Davis, K. C. Blanchard, *J. Am. Chem. Soc.* **1929**, *51*, 6, 1790–1801.

[10] T. L. Lemke, *Review of Organic Functional Groups: Introduction to Medicinal Organic Chemistry,* Lea and Febiger, Philadelphia, **1983**.

[11] M. Eckert-Maksić, H. Maskill, I. Zrinski, *J. Chem. Soc., Perkin Trans*. 2. **2001**, 2147–2154.

[12] C. A. Marlies, V. K. La Mer, *J. Am. Chem. Soc.* **1935**, *57*, 1812–1820.

[13] C. H. Arrowsmith, A. Awwal, B. A. Euser, A. J. Kresge, P. P. T. Lau, D. P. Onwood, Y. C. Tang, E. C. Young, *J. Am. Chem. Soc.* **1991**, *113*, 172–179.

[14] C. H. Arrowsmith, A. J. Kresge, Y. C. Tang, *J. Am. Chem. Soc.* **1991**, *113*, 179–182.

[15] R. A. Cox, *Can. J. Chem.* **1996**, *74*, 1774–1778.

[16] Gaussian 16, Revision C.01, M. J. Frisch, G. W. Trucks, H. B. Schlegel, G. E. Scuseria, M. A. Robb, J. R. Cheeseman, G. Scalmani, V. Barone, G. A. Petersson, H. Nakatsuji, X. Li, M. Caricato, A. V. Marenich, J. Bloino, B. G. Janesko, R. Gomperts, B. Mennucci, H. P. Hratchian, J. V. Ortiz, A. F. Izmaylov, J. L. Sonnenberg, D. Williams-Young, F. Ding, F. Lipparini, F. Egidi, J. Goings, B. Peng, A. Petrone, T. Henderson, D. Ranasinghe, V. G. Zakrzewski, J. Gao, N. Rega, G. Zheng, W. Liang, M. Hada, M. Ehara, K. Toyota, R. Fukuda,

J. Hasegawa, M. Ishida, T. Nakajima, Y. Honda, O. Kitao, H. Nakai, T. Vreven, K. Throssell, J. A. Montgomery, Jr., J. E. Peralta, F. Ogliaro, M. J. Bearpark, J. J. Heyd, E. N. Brothers, K. N. Kudin, V. N. Staroverov, T. A. Keith, R. Kobayashi, J. Normand, K. Raghavachari, A. P. Rendell, J. C. Burant, S. S. Iyengar, J. Tomasi, M. Cossi, J. M. Millam, M. Klene, C. Adamo, R. Cammi, J. W. Ochterski, R. L. Martin, K. Morokuma, O. Farkas, J. B. Foresman, and D. J. Fox, D. J. Gaussian, Inc., Wallingford CT, **2016**.

[17] G. te Velde, F. M. Bickelhaupt, E. J. Baerends, C. Fonseca Guerra, S. J. A. van Gisbergen, J. G. Snijders, T. Ziegler, *J. Comp. Chem.* **2001**, *22*, 931–967.

[18] ADF 2019.3, SCM, Theoretical Chemistry, Vrije Universiteit, Amsterdam, The Netherlands, http://www.scm.com.

[19] J. P. Perdew, K. Burke, M. Ernzerhof, *Phys. Rev. Lett.* **1996**, *77*, 3865–3868; erratum **1996**, *78*, 1396.

[20] C. Adamo, V. Barone, *J. Chem. Phys.* **1999**, *110*, 6158–6170.

[21] M. Ernzerhof, G. E. Scuseria, *J. Chem. Phys.* **1999**, *119*, 5029–5036.

[22] F. Weigend, R. Ahlrichs, *Phys. Chem. Chem. Phys.* **2005**, *7*, 3297–3305.

[23] S. Grimme, J. Antony, S. Ehrlich, H. Krieg, *J. Chem. Phys.* **2010**, *132*, 154104.

[24 S. Grimme, S. Ehrlich, L. Goerigk, *J. Comp. Chem.* **2011**, *32*, 1456–1465.

[25] P. Pulay, J. F. Hinton, in *Encyclopedia of Nuclear Magnetic Resonance* (Eds.: D. M. Grant, R. K. Harris), Volume 7, Wiley, New York, **1996**, pp. 4334–4339.

[26] A. D. Becke, *Phys. Rev. A*, **1988**, *38*, 3098–3100.

[27] J. P. Perdew, *Phys. Rev. B*, **1986**, *33*, 8822–8824 erratum **1986**, *34*, 7406.

[28] E. van Lenthe, E. J. Baerends, *J. Comp. Chem.* **2003**, *24*, 1142–1156.

[29] E. van Lenthe, E. J. Baerends, J. G. Snijders, *J. Chem. Phys.* **1994**, *101*, 9783.

[30] C. J. Jameson, A. K. Jameson, D. Oppusunggu, S. Wille, P. M. Burrell, J. Mason, *J. Chem. Phys.* **1981**, *74*, 81–88.

[31] M. Mitoraj, A. Michalak, T. Ziegler, *J. Chem. Theory Comput.* **2009**, *5*, 962–975.

[32] J. S. Anderson, V. M. Iluc, G. L. Hillhouse, *Inorg. Chem.* **2010**, *49,* 10203–10207.

[33] R. Beck, M. Shoshani, J. Krasinkiewicz, J. A. Hatnean, S. A. Johnson, *Dalton Trans.* **2013**, *42*, 1461-1475.

[34] Y.-E. Kim, J. Kim, Y. Lee, *Chem. Commun.* **2014**, *50*, 11458–11461.

[35] S. Gambarotta, C. Floriani, A. Chiesi-Villa, C. Guastini, *J. Am. Chem. Soc.* **1985**, *107*, 2985-2986.

[36] J. A. Buss, D. G. Vandervelde, T. Agapie, *J. Am. Chem. Soc.* **2018**, *140*, 10121–10125.

[37] R. G. Carden, J. J. Ohane, R. D. Pike, P. M. Graham, *Organometallics* **2013**, *32*, 2505–2508.

[38] L. Contreras, M. Paneque, M. Sellin, E. Carmona, P. J. Perez, E. Gutierrez-Puebla, A. Monge, C. Ruiz, *New J. Chem.* **2005**, *29*, 109-115.

[39] G. S. Bristow, P. B. Hitchcock, M. F. Lappert, *J. Chem. Soc., Chem. Commun.* **1981**, 1145–1146.

[40] P. F. Fu, M. A. Khan, K. M. Nicholas, *J. Am. Chem. Soc.* **1992**, *114*, 6579–6580.

[41] S. Komiya, M. Akita, N. Kasuga, M. Hirano, A. Fukuoka, *J. Chem. Soc., Chem. Commun.* **1994**, 1115–1116.

[42] T.-F. Wang, C.-C. Hwu, C.-W. Tsai, K.-J. Lin, *Organometallics* **1997**, *16*, 3089–3090.

[43] T.-F. Wang, C.-C. Hwu, Y.-S. Wen, *J. Organomet. Chem.* **2004**, *689*, 411–418.

[44] W. L. Earl, D. L. VanderHart, *J. Magn. Reson*. **1982**, *48*, 35–54.

[45] P. Bertani, J. Raya, B. Bechinger, *Solid State Nucl. Magn. Reson*. **2014**, *61–62*, 15–18.

[46] M. Witanowski, L. Stefaniak, S. Szymański, H. Januszewski, *J. Magn. Reson*. **1977**, *28*, 217–226.

[47] M. Witanowski, G. A. Webb, Eds., *Nitrogen NMR*, Plenum Press, London and New York, **1973**.

[48] a) M. Witanowski, G. A. Webb, *Annu. Rep. NMR Spectrosc.* **1972**, *5A*, 395-464; b) M. Witanowski, L. Stefaniak, G. A. Webb, *Annu. Rep. NMR Spectrosc.* **1978**, *5*, 117-244; c) M. Witanowski, L. Stefaniak, G. A. Webb, *Annu. Rep. NMR Spectrosc.* **1981**, *11B*, 1-486; d) M.

Witanowski, L. Stefaniak, G. A. Webb, *Annu. Rep. NMR Spectrosc.* **1987**, *18B*, 1-211; e) M. Witanowski, L. Stefaniak, G. A. Webb, *Annu. Rep. NMR Spectrosc.* **1993**, *25*, 1-82.

49 P. R. Srinivasan, R. L. Lichter, *J. Magn. Reson*. **1977**, *28*, 227–234.

50 G. C. Levy, R. L. Lichter, *Nitrogen-15 Nuclear Magnetic Resonance Spectroscopy*, Wiley-Interscience, John Wiley & Sons, New York, **1979**.

51 R. K. Harris, E. D. Becker, S. M. Cabral de Menezes, R. Goodfellow, P. Granger, *Pure Appl. Chem.* **2001**, *73*, 1795–1818.

52 T. Duncan, *Principal Components of Chemical Shift Tensors: A Compilation*, 2nd Ed, The Farragut Press, Chicago, **1994**.

53 R. K. Harris, R. E. Wasylishen, Eds., *Encyclopedia of Nuclear Magnetic Resonance*, Volume 5, John Wiley & Sons, Ltd., Chichester, **2012**.

54 J. Mason, L. F. Larkworthy, E. A. Moore, *Chem. Rev.* **2002**, *102*, 913–934.

55 W. von Philipsborn, R. Müller, *Angew. Chem., Int. Ed. Engl.* **1986**, *25*, 383–413; *Angew. Chem.* **1986**, *98*, 381–412.

56 S. Hayashi, K. Hayamizu, *Bull. Chem. Soc. Jpn*. **1991**, *64*, 688–690.

57 M. Dračínský, P. Bouř, *J. Comput. Chem.* **2012**, *33*, 1080–1089.

58 L. Stievano, F. Tielens, I. Lopes, N. Folliet, C. Gervais, D. Costa, J.-F. Lambert, *Cryst. Growth Des.* **2010**, *10*, 3657–3667.

59 P. K. Bhattacharyya, B. P. Dailey, *J. Chem. Phys.* **1973**, *59*, 5820–5823.

60 Y. A. Strelenko, N. M. Sergeyev, *J. Mol. Structure*, **1996**, *378*, 61–65.
